# An Arduino-Based Butane Leak Detection System With Automated Ventilation and Internet of Things (IoT) Mobile Application

**DOI:** 10.7759/cureus.116225

**Published:** 2026-09-14

**Authors:** Rayyan Khan, Noha Khan, Izyan Khan, Sharath Sanjay, Zaid Ahmad, Imteyaz Khan

**Affiliations:** 1 Simulation, Bayonne School District, Bayyone, USA; 2 Simulation, County Prep High School, Jersey City, USA; 3 Simulation, West Windsor-Plainsboro Regional School District, Princeton Junction, USA; 4 Simulation, Heidelberg University, Heidelberg, DEU; 5 Neonatology, Saint Peter's University Hospital, New Brunswick, USA

**Keywords:** arduino microcontroller, automated ventilation, gas leak detection, internet of things (iot), mq gas sensors

## Abstract

Residential gas leaks remain a widespread household safety concern worldwide. These leaks can cause explosions, air pollution, and fires, which result from incomplete combustion. Incomplete combustion produces a by-product, that is, carbon monoxide, which can spread in a matter of minutes and reach high concentrations in seconds. Conventional preventive measures, such as manual regulators and periodic maintenance, are insufficient against sudden hose ruptures or regulator failures. This experiment validates a solution to this problem of harmful gas accumulation by utilizing a combined approach of prompt detection and elimination of leaked gas using MQ gas sensors, automatic servo-controlled exhaust fans, and automated window actuators. The testing included a standard pressurized butane gas canister, which was sprayed onto the MQ-6 sensor as well as the MQ-4 and MQ-2 gas sensors in a controlled environment. An Arduino Wi-Fi microcontroller (Monza, Italy) was used to integrate Wi-Fi connectivity with the countermeasures, which consisted of a DC motor-powered fan, as well as a light-emitting diode (LED) and a buzzer. These were used to mitigate any additional effects of the gas leak. The system components were installed within and along the frame of a model house to simulate a residential environment. To test the system, a pressurized can of butane was used to simulate the conditions of air quality after a gas leak. Results clearly indicated that the MQ gas sensors were reliable gas sensors for detecting gas leaks and the integration of both physical countermeasures and an online-based Internet of Things (IoT) alarm system was effective in warning of gas leaks and mitigating any additional effects.

## Introduction

Background

As per the National Fire Protection Association, annually, approximately 125,000 accidents happen in the United States related to cooking gas, and according to the Global Burn Registry (GBR), 25% of individuals sustain cooking-related burns, with 22% being cookstove-related burns [1]. As per the World Health Organization, roughly 265,000 annual deaths stem from burns worldwide [2]. Gas leaks occur when the fuel supplied to cooking stoves leaks, often caused by faulty flexible connectors, poor installation, or aging, corroded pipes, causing explosions and deadly gases to be released. Due to this, raw butane can be released into the air and cause household air pollution, displacing the oxygen in the air and causing asphyxiation. Another consequence is that the incomplete combustion of gas creates carbon monoxide. In a small area such as a residential kitchen, carbon monoxide, which is colorless and odorless, can be inhaled by unsuspecting residents and cause carbon monoxide poisoning. These can leave lasting effects, some of which are fatal [3]. According to the World Health Organization, leaking, cooking, and heating fuels cause 2.9 million injuries and fatalities every year through household air pollution [4]. In times of crisis, conditions like these make it harder to escape. Normal gas leak detectors will sound an alarm when there is a gas leak or fire but are useless if occupants are not home [5]. This is the main reason the project was designed because the system not only detects early gas leaks but also can remove gas from the kitchen via an exhaust fan and remotely alert people on their phones who are not at home. The proposed system utilizes MQ gas detectors to detect butane gas (LPG) in the resident's house. When the gas sensors detect a change in air quality, they will trigger the system by using the Arduino UNO R4 Wi-Fi microcontroller (Monza, Italy) to activate two individual motors, one of which turns on the exhaust fan and the other opens the window of the user's house, which combined serves as the automated ventilation. Buzzers and light-emitting diodes (LEDs) will also activate to warn anyone currently residing in the house of the change in air quality. After the precautions are taken, the Arduino UNO R4 Wi-Fi sends notifications immediately to the user through the Internet of Things (IoT) app. The app allows the user to control the exhaust and windows, as well as view a live feed from the MQ gas sensor.

Purpose

This paper presents the design, development, and validation of a low-cost, microcontroller-based smart safety system capable of real-time detection of butane leakage, immediate audio-visual alarm generation, automated mechanical ventilation through servo-assisted window opening and forced-air exhaust, and instantaneous remote notification via an IoT platform. The prototype, built around an Arduino UNO R4 Wi-Fi microcontroller and an MQ gas sensor, demonstrates reliable operation at a total component cost below $130, making it suitable for widespread adoption in a resource-constrained household. This work addresses the identified safety gap by engineering an active, multi-layered mitigation system that not only detects hazardous concentrations of butane but also executes immediate physical countermeasures and informs occupants irrespective of their location, unlike most standard carbon monoxide or gas detectors which are purely alert systems, requiring people nearby to take immediate action themselves.

The proposed system was designed to fulfill the following functional requirements: early detection of butane (primarily butane present in a pressurized can) at concentrations well below the lower explosive limit (1.6%) [6], generation of high-decibel audible and high-visibility visual alarms within two seconds of detection, autonomous initiation of mechanical ventilation via window actuation and activation of an exhaust fan to reduce indoor gas concentration, and transmission of real-time alerts to the user's smartphone through a secure IoT framework, enabling response even when residents are away from the premises.

## Technical report

Materials and methods 

Hardware

For the hardware setup, the project runs on (1) an Arduino UNO R4 Wi-Fi microcontroller board. The detection and alert system utilizes (2) MQ gas sensors, (3) a buzzer, and (4) LEDs, while (5) a servo motor and (6) a DC motor-powered exhaust fan handle the automated physical response. (7) A DHT-11 heat sensor was used to measure the temperature during the test run to ensure the consistency of results as the MQ sensors are affected by temperature. All of these components are housed inside (8) a model kitchen and connected using a breadboard adapter and a 5V, 2A power cable, as seen in Figures 1-2. To safely test the system, a standard pressurized butane canister is used to simulate gas leaks.

**Figure 1:**
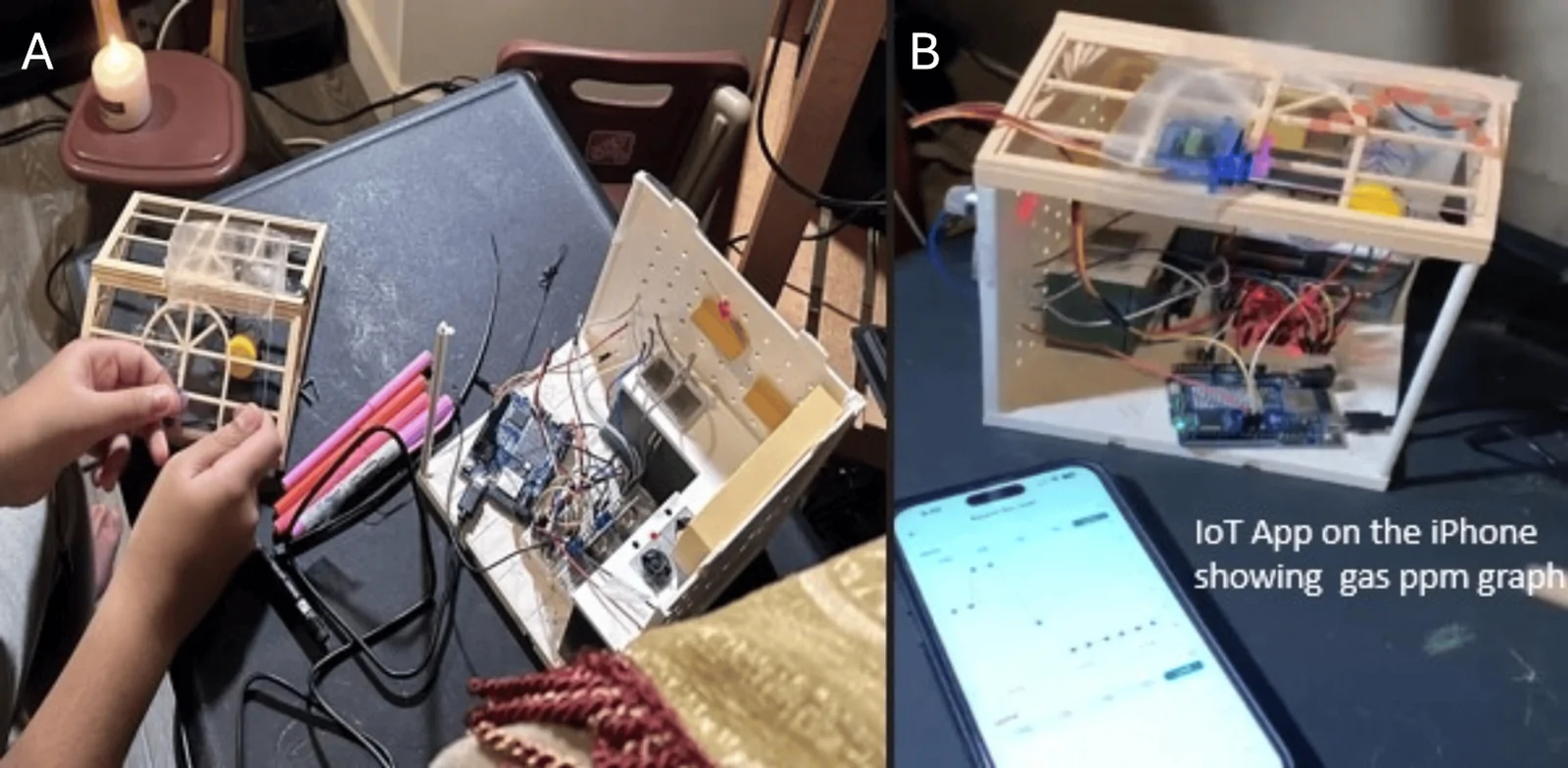
Structural prototyping phase and final integration of the Internet of Things gas detection system (A) The structural housing construction, highlighting the initial alignment of the Arduino microcontroller unit, sensor wiring, and hardware components within the model kitchen frame. (B) Fully assembled prototype in operational mode, featuring the integrated internal electronic circuitry alongside the synchronized mobile application interface. The smartphone display tracks real-time analog data streams and sensor readings calibrated in parts per million (ppm).

**Figure 2:**
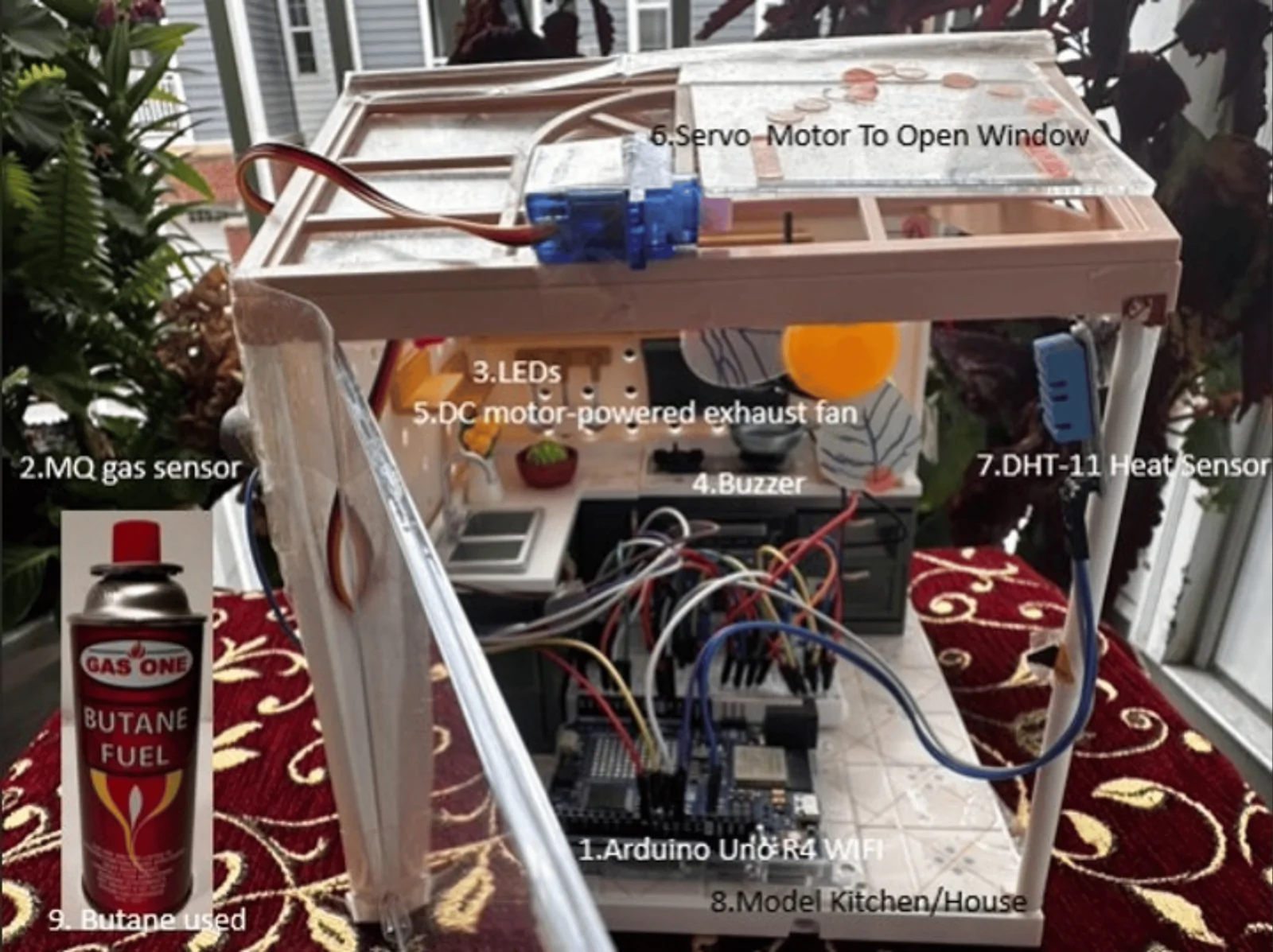
Fully assembled hardware prototype configuration featuring active physical mitigation components The constructed experimental model highlights the spatial layout of all numbered system parts: (1) Arduino UNO R4 Wi-Fi microcontroller board, (2) MQ gas sensor probe, (3) indicator light-emitting diodes (LEDs), (4) active buzzer, (5) DC motor-powered exhaust fan, (6) micro servo motor for automated window actuation, (7) DHT-11 ambient temperature sensor, (8) scaled model kitchen enclosure frame, and (9) pressurized butane fuel canister utilized for simulation testing.

The following are the specifications of the components: Arduino UNO R4 Wi-Fi microcontroller (official SKU: ABX00087; Arduino, Monza, Italy), main microcontroller (model: RA4M1 series (R7FA4M1AB3CFM#AA0); Renesas, Tokyo, Japan), processor (48 MHz Arm Cortex-M4 with floating-point unit), secondary MCU (Wi-Fi/Bluetooth) (model: ESP32-S3-MINI-1-N8; Espressif, Shanghai, China), breadboard (item model number: EL-CP-028; product model: 6PCS 400 Point Breadboard Kit; ELEGOO, Shenzhen, China), servo motor (DIANN model/item number: DI09512; product model: SG90 9G), gas sensor (UMLIFE Mfr part number: MQ-6; main chip: LM393 with MQ-x gas sensing probe), DHT-11 (digital temperature and humidity sensor; item model number: 3-01-0039-1; part number: 3-01-0039-A; HiLetgo, Shenzhen, China), buzzer (item model number: 3-01-1674; product type: active integrated buzzer/alarm sounder; HiLetgo, Shenzhen, China), fan DC motor (voltage: 3-6V; speed (at 3V): 19,000 RPM; SunFounder Elite Explorer Kit, Wilmington, Delaware, United States), and red LED (generic type: 5 mm standard diffused LED; 2-3.2V forward voltage; 20mA current rating).

Software

The firmware was crafted in the Arduino IDE using C++. Connecting to the Arduino IoT platform via Wi-Fi provides notifications, event logging, and remote monitoring, eliminating the need for static IP addresses or port forwarding. Threshold calibration was performed empirically in a controlled environment using fresh air close to a high-efficiency particulate air (HEPA) and gas filter, following standard baseline metric guidelines. The code used in the project has been supplied as Appendix A. The circuit diagram is shown in Figure 3.

**Figure 3:**
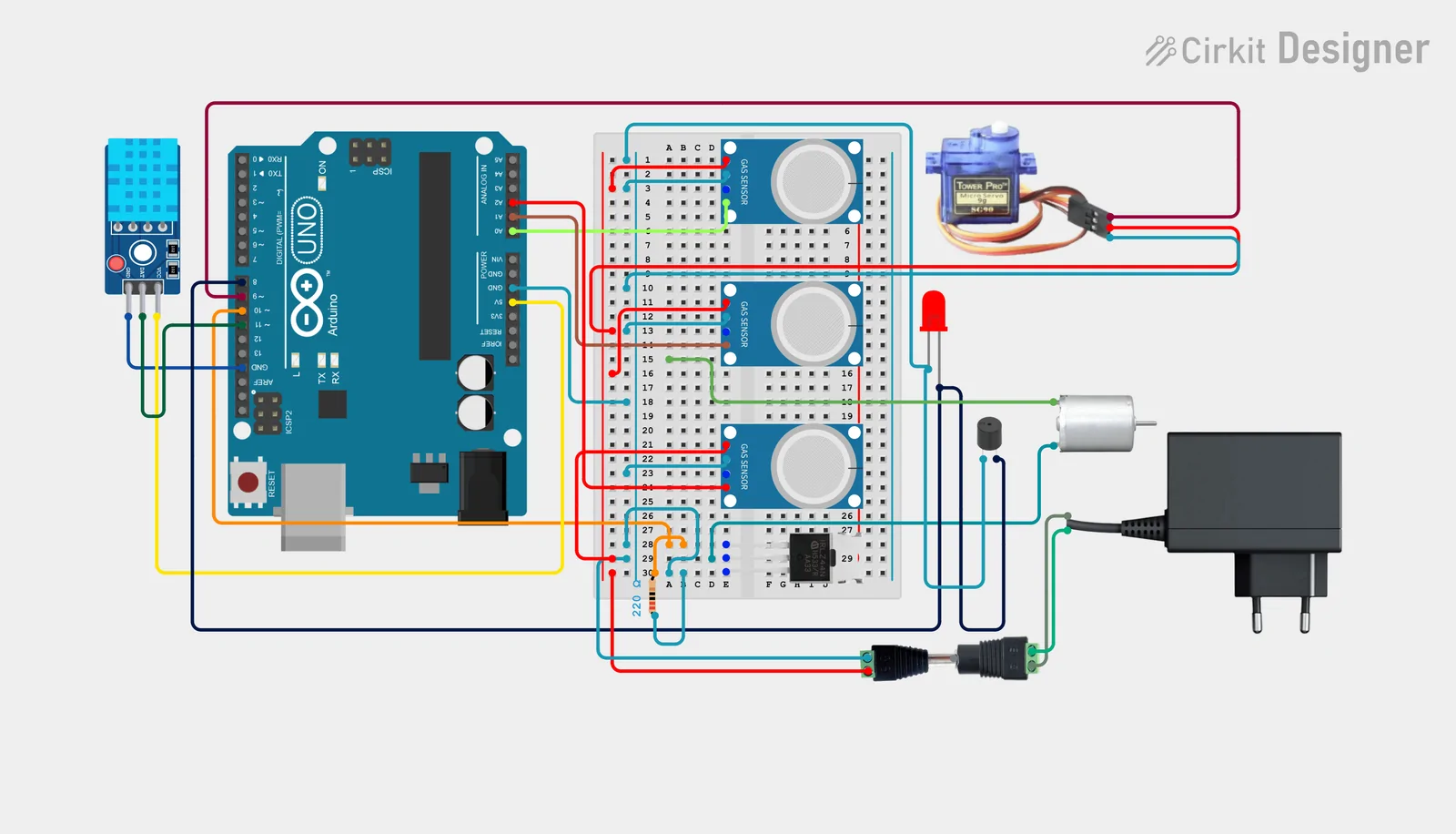
Schematic circuit diagram illustrating the component wiring matrix for the gas detection system This layout details the electrical connections linking the Arduino microcontroller unit to the MQ-6, MQ-4, and MQ-2 analog gas sensors, a light-emitting diode (LED), an active buzzer, a 5V/2A DC power supply, a 3-6V DC ventilation motor, an SG90 micro servo motor, and a DHT-11 temperature and humidity sensor. This schematic diagram was developed manually by the authors using Cirkit Designer (Cirkit Design LLC, Lower Gwynedd, Pennsylvania, United States).

Methodology

The proposed device was developed using common components for accessibility. The device is powered by a power cable connected to a 5V battery, which is plugged into the USB-C port on the Arduino UNO R4 Wi-Fi microcontroller, from which the MQ gas sensors and motors were attached. The buzzer and LED were put on the breadboard, with the 5V 2A cable plugged into the MQ gas sensors. The DC and servo motors were set to activate once the Arduino sensed an electric charge sent by the MQ gas sensors. The servo motor would open the window, and the DC motor would start the ventilation fan. An LED and buzzer would also activate for additional safety measures. The MQ gas sensor was chosen specifically for this project due to it being a semiconductor gas sensor sensitive to butane, propane, methane (natural gas), hydrogen, alcohol, smoke, etc. [7].

The MQ sensor uses a tin dioxide (SnO₂)-sensitive layer whose resistance changes in the presence of abnormal gases. In clean air, SnO₂ has high resistance. When flammable gas or smoke is present, the gas molecules reduce the surface, decreasing resistance [8]. This change in resistance is measured as an output voltage, typically through a simple voltage divider circuit. When the MQ-6 gas sensor measured a voltage change of 3.53V due to butane gas in the air, it correlated to 500 ppm butane, and this was set as the trigger for the alarm. This spike is the current that gets sent to the Arduino UNO R4 Wi-Fi microcontroller. This setup was placed in a frame set to model a miniature house, so that it may mimic an environment where gas leaks are most common. The system was set on the ceiling, with panels to represent the windows. An example of this is shown in Figure 2. A pressurized butane canister was used to mimic poisonous and flammable vapors commonly found in gas leaks [9]. This was all set up with an IoT online app, which not only showed the voltage outputs of the MQ gas sensors but also could turn on and off both motors and send a notification based on the MQ sensor readings. Video 1 demonstrates how this project works in real-time. The app is showcased in Figure 4, as well as the live readings of the MQ gas sensors.

**Video 1:** Demonstration of the project working in real-time

**Figure 4:**
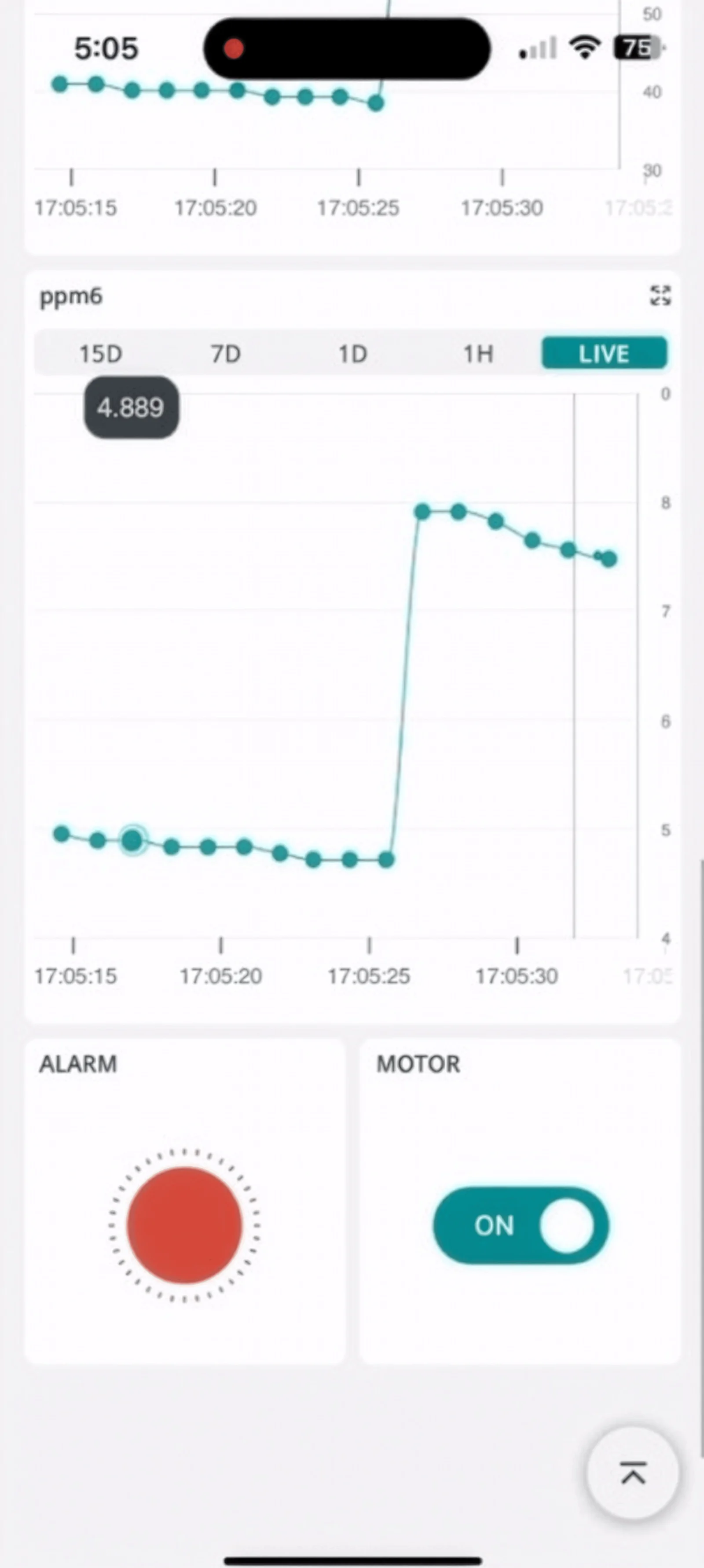
Real-time user interface display of the integrated Internet of Things mobile application dashboard The application interface tracks live sensor data streams during an experimental trial, displaying a sharp real-time voltage spike in the parts per million (ppm) telemetry reading of the MQ-6 gas sensor (ppm6) concurrently with active alarm state indicators and automated ventilation motor deployment controls.

Safety Threshold and Response Logic

The system's alarm limit was set conservatively at 1500 (raw analog-to-digital converter (ADC) value, corresponding to approximately 500-1000 ppm or less than 6.25% of the lower explosive limit) to facilitate a timely response and prevent false triggers from cooking fumes. Once triggered, the alarm system initiates a cycle of flashing lights and buzzer alerts every 100 milliseconds (ms), simultaneously engaging a relay to power the exhaust fan and commanding a servo to open the window, completing these actions in under 1.8 seconds. An HTTP POST request sends a push notification over 2.4 GHz Wi-Fi within 2-4 seconds. The system draws 180 mA while idle and up to 780 mA during a full alarm state. During a 72-hour baseline test, the false-positive rate was held under 2%.

Results

To test this, 11 tests were run with butane. One minute was given for calibration before the butane was sprayed. Two minutes were given after to collect results and let the sensors rest. The DHT-11 heat and humidity sensor was used to ensure consistent temperatures during the test run as temperature can affect the MQ sensor readings. The average temperature during the test was 23.7°C. The average humidity during the test was 55.92% which is generally considered comfortable and standard for most testing environments, indoor spaces, and laboratory conditions. The analog data of the 11 test runs have been supplied as Appendix B in Excel format. The results are displayed as a graph in Figure 5.

**Figure 5:**
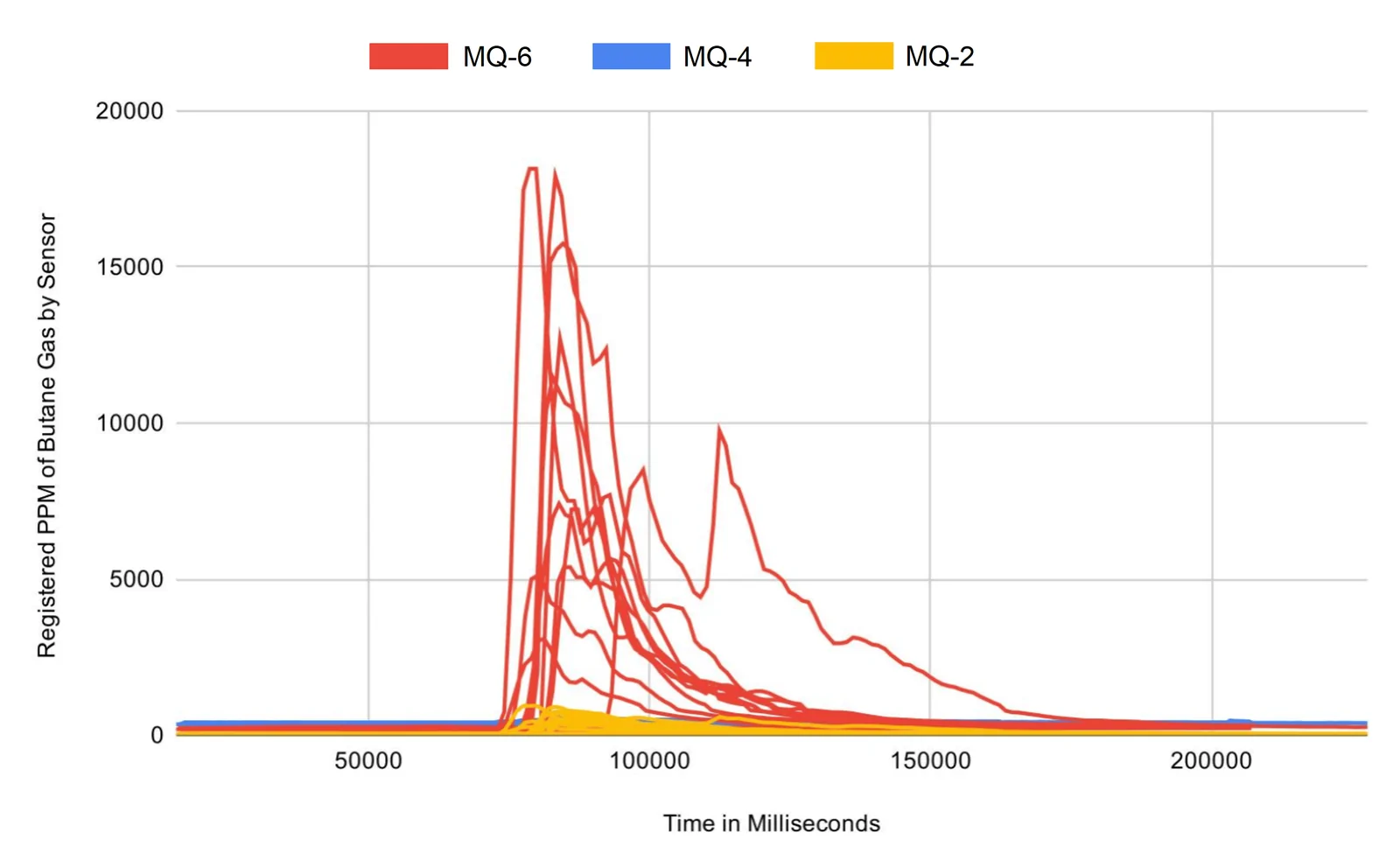
Graphic analytical evaluation of sensor voltage responses across the 11 butane exposure trials Upon initial gas introduction, the sensor demonstrated a steep, non-linear voltage increase from the baseline voltage. The mean response time required to reach 90% of the maximum voltage peak was 1.2 seconds, reflecting rapid chemical adsorption on the active sensing layer.

We used the paired t-test to compare baseline and post-exposure readings, which indicated a statistically significant response to butane (p=0.01). This confirms that the sensors reliably detected the gas. There is also consistency visible in the overlapping rise and decay slopes across the 11 trials in Figure 5. Readings fell back below the alarm threshold in 43.15 seconds. The alarm triggered within 1.2 seconds of exposure to 1000 ppm of target gas. At a one-meter range, the audible alarm measured between 104 and 108 dB(A). The automated windows reliably opened in approximately one second. The Arduino IoT Cloud application also provided real-time monitoring of gas levels.

## Discussion

This research project aimed to demonstrate the viability of a low-cost, active mitigation system for residential gas leaks using an MQ gas sensor, an Arduino UNO R4 Wi-Fi microcontroller, and automatic ventilation. The data from the 11 test trials revealed a highly significant response to changes in air quality (p=0.01). With a detection latency of less than 1.2 seconds upon the introduction of 1000 ppm of pressurized butane and a mean overall response time of 43.15 seconds to execute the ventilation protocol, the system proves highly capable of intervening before gas concentrations reach hazardous levels. When a pressurized butane canister was sprayed near the MQ gas sensors, the graph recorded a voltage spike, indicating a drop in resistance. This spike sent an electrical current to the microcontroller, prompting the Arduino to activate the system. Upon activation, both the DC motor and servo motor began operating, initiating the automated ventilation. Concurrently, the alarms and buzzer activated, continuing as long as the MQ gas sensor detected a change in air quality. Once the system completed its cycle, the app sent a notification about the air quality change. The graph displayed the MQ gas sensor's real-time readings in parts per million (ppm). Unlike conventional gas detectors that rely solely on passive alarms, this system bridges a critical safety gap by initiating immediate physical countermeasures, that is, exhausting contaminated air and opening ventilation, while simultaneously alerting users remotely.

However, this study has some limitations that must be acknowledged. First, the testing was conducted in a scaled-down model kitchen rather than a full-sized residential environment. The 43.15-second clearance time will likely scale differently in a larger room depending on the cubic footage and the power of the exhaust fans used. Second, the system was validated exclusively using pressurized butane; while the MQ sensor is broadly sensitive to other combustible gases such as methane and propane, response times for those specific gases were not empirically tested in this study. Finally, the long-term reliability of the MQ sensor's SnO₂ layer, which can be susceptible to degradation or drift over months of continuous use in varying humidity, was not evaluated.

We acknowledge that gas detection systems carry significant responsibility, as false negatives or delayed alerts could lead to dangerous situations. It is important to emphasize proper calibration, regular testing, fail-safe mechanisms, and clear user warnings to ensure the system prioritizes safety.

## Conclusions

The prototype successfully fulfilled its design objectives by providing automated ventilation and active remote awareness. Simultaneously, the DC motor engaged the exhaust fan, while the servo motor opened the ventilation panel. After all the precautions had been completed, the notification was sent to the phone through the app about the change in air quality, with a live feed from the MQ gas sensors. The MQ gas sensors played a major role in this project with their SnO₂ resistance to natural air, which allowed for sensing changes in air quality through voltage. This feature allowed the microcontroller to turn on the automated ventilation using voltage signals that the app can read. The application also provides real-time data, so users can monitor gas concentrations from afar. Once this voltage exceeds a set safety limit, the microcontroller activates its safety protocol. It triggers the exhaust fans to remove the contaminated air and sends an instant alert to the user's device. This smooth interaction between hardware and software creates a quick-response safety system, making sure that harmful gases are effectively removed from the environment before they become dangerous.

Worldwide, many burn patients suffer significant morbidity and mortality due to the severity of burns from cooking gas explosions in kitchens and also due to a lack of adequate care in resource-limited countries. So, it is imperative to find innovative solutions to prevent these burns from happening. We believe this innovation of timely notification of gas leaks and an integrated solution for actively getting rid of explosive and poisonous gas will go a long way in decreasing the burn burden in society and transforming healthcare.

## Figures and Tables

**Figure 6:**
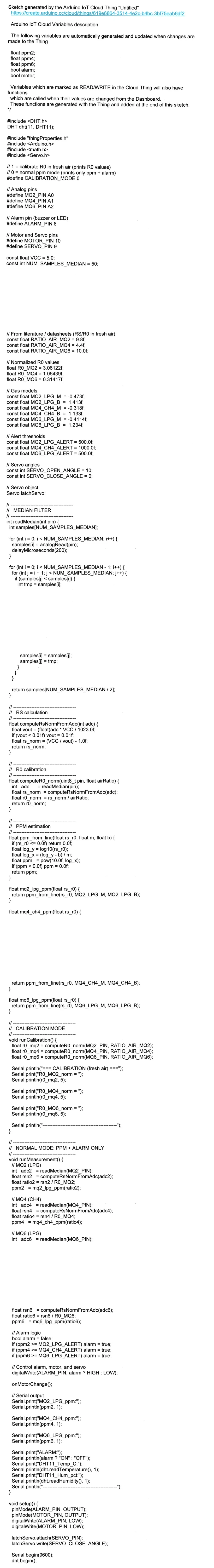
Code used in the project

## Appendices

Appendix A

Appendix B

**Table 1:** Analog data of the 11 test runs with butane

MQ2_ppm	MQ6_Butane_ppm	MQ4_CH4_ppm	ALARM_bool	DHT11_Temp_C	DHT11_Hum_pct	time_ms
Test run 1						
352	237.2	46.5	OFF	23.3	52.6	16089
352	237.2	46.5	OFF	23.3	52.6	17207
346.8	237.2	46.1	OFF	23.5	55.9	18346
346.8	237.2	46.5	OFF	23.5	55.9	19463
346.8	237.2	46.1	OFF	23.5	55.9	20602
346.8	240.8	46.5	OFF	23.5	55.9	21720
346.8	240.8	47.4	OFF	23.5	56	22860
346.8	248.2	47.9	OFF	23.5	56	23978
346.8	248.2	47.9	OFF	23.5	56	25118
357.2	252	48.3	OFF	23.5	56	26236
346.8	255.8	49.3	OFF	23.5	56	27376
346.8	259.7	49.7	OFF	23.5	56	28493
346.8	259.7	49.7	OFF	23.5	56	29632
346.8	263.6	50.2	OFF	23.5	56	30755
346.8	267.6	50.7	OFF	23.5	56.1	31900
346.8	267.6	51.2	OFF	23.5	56.1	33018
346.8	271.6	51.7	OFF	23.5	56.1	34156
346.8	275.6	51.7	OFF	23.5	56.1	35274
346.8	275.6	51.7	OFF	23.5	56.1	36413
346.8	275.6	51.7	OFF	23.5	56.1	37531
346.8	279.8	52.2	OFF	23.5	56.1	38670
346.8	275.6	51.7	OFF	23.5	56.1	39788
346.8	275.6	52.2	OFF	23.6	56	40926
346.8	275.6	52.2	OFF	23.6	56	42044
352	275.6	51.7	OFF	23.5	56.1	43183
346.8	279.8	51.7	OFF	23.5	56.1	44301
346.8	275.6	51.7	OFF	23.6	56.1	45439
346.8	271.6	51.7	OFF	23.6	56.1	46557
352	271.6	51.2	OFF	23.5	56.1	47696
346.8	271.6	51.2	OFF	23.5	56.1	48814
346.8	271.6	51.2	OFF	23.6	56	49955
346.8	271.6	51.7	OFF	23.6	56	51073
346.8	271.6	51.2	OFF	23.6	56.1	52211
341.7	271.6	51.2	OFF	23.6	56.1	53329
346.8	275.6	51.7	OFF	23.6	56.1	54466
346.8	275.6	51.7	OFF	23.6	56.1	55583
346.8	279.8	52.2	OFF	23.6	56.1	56721
346.8	279.8	52.2	OFF	23.6	56.1	57839
346.8	283.9	52.2	OFF	23.6	56.1	58978
341.7	283.9	52.2	OFF	23.6	56.1	60096
346.8	288.2	52.7	OFF	23.6	56.1	61239
346.8	283.9	52.7	OFF	23.6	56.1	62362
346.8	283.9	52.7	OFF	23.6	56	63501
346.8	279.8	52.2	OFF	23.6	56	64619
341.7	275.6	51.7	OFF	23.6	56	65760
341.7	275.6	51.7	OFF	23.6	56	66878
341.7	271.6	51.2	OFF	23.6	56	68019
346.8	267.6	50.7	OFF	23.6	56	69137
341.7	263.6	50.2	OFF	23.6	56	70277
341.7	259.7	49.3	OFF	23.6	56	71395
341.7	255.8	49.3	OFF	23.6	55.9	72533
341.7	252	48.3	OFF	23.6	55.9	73651
341.7	252	48.3	OFF	23.6	55.9	74789
341.7	248.2	47.9	OFF	23.6	55.9	75907
341.7	244.5	47.9	OFF	23.6	56	77046
341.7	244.5	47.4	OFF	23.6	56	78164
341.7	244.5	47.9	OFF	23.6	55.9	79305
395.3	244.5	47.4	OFF	23.6	55.9	80423
406.7	248.2	50.7	OFF	23.6	56	81562
412.4	1408.2	177.6	ON	23.6	56	82682
486.3	3630.6	367.5	ON	23.6	55.9	83822
486.3	5584	490.8	ON	23.6	55.9	84942
479.8	7235.5	563.7	ON	23.6	55.9	86082
473.4	7235.5	538.1	ON	23.6	55.9	87201
473.4	6162.4	496.5	ON	23.6	55.9	88340
467	6316.3	538.1	ON	23.6	55.9	89459
467	6972	550.7	ON	23.6	55.9	90598
460.7	7602.7	570.3	ON	23.6	55.9	91723
460.7	7697.5	550.7	ON	23.6	55.9	92868
460.7	6801.8	513.8	ON	23.6	55.9	93988
460.7	5866	474.4	ON	23.6	56	95129
460.7	5723.3	453.4	ON	23.6	56	96249
454.5	5123.1	405.6	ON	23.6	56	97389
454.5	4366.2	363.5	ON	23.6	56	98507
454.5	4006.1	355.7	ON	23.6	55.9	99647
454.5	3813.8	348.1	ON	23.6	55.9	100766
454.5	3413.8	319.4	ON	23.6	56	101906
454.5	3017.8	299.7	ON	23.6	56	103026
448.3	2634.2	278.4	ON	23.6	55.9	104167
448.3	2269.7	258.7	ON	23.6	55.9	105288
448.3	2132.8	245.7	ON	23.6	55.9	106429
448.3	1954.3	231	ON	23.6	55.9	107548
448.3	1767.8	210.7	ON	23.6	56	108691
448.3	1598.4	196.3	ON	23.6	56	109810
448.3	1481.6	188.6	ON	23.6	56	110951
442.2	1500.5	190.5	ON	23.6	56	112071
442.2	1462.9	186.7	ON	23.6	56	113212
442.2	1355.5	175.8	ON	23.6	56	114331
442.2	1271.7	174	ON	23.6	56	115472
436.1	1288	183	ON	23.6	56	116591
436.1	1372.9	194.3	ON	23.6	56	117733
436.1	1408.2	194.3	ON	23.6	56	118853
436.1	1408.2	190.5	ON	23.6	55.9	119994
436.1	1355.5	181.1	ON	23.6	55.9	121114
436.1	1255.5	172.3	ON	23.6	56	122261
436.1	1162.5	164	ON	23.6	56	123386
436.1	1075.9	156.1	ON	23.6	56	124528
430.1	1021.6	150	ON	23.6	56	125648
430.1	995.4	147.1	OFF	23.6	55.9	126790
357.2	796.4	122.2	OFF	23.6	55.9	127911
357.2	796.4	121	OFF	23.6	55.9	129053
352	775.6	118.7	OFF	23.6	55.9	130174
357.2	755.2	116.4	OFF	23.6	55.9	131317
352	735.4	115.3	OFF	23.6	55.9	132438
352	735.4	114.2	OFF	23.6	55.9	133580
352	725.6	113.1	OFF	23.6	55.9	134701
352	706.5	109.8	OFF	23.6	55.9	135843
352	669.6	105.7	OFF	23.6	55.9	136963
352	634.4	101.7	OFF	23.6	55.9	138103
352	601	97.8	OFF	23.6	55.9	139223
352	569.1	95.1	OFF	23.6	55.9	140362
352	538.8	91.5	OFF	23.6	55.9	141482
352	517	88.9	OFF	23.6	55.9	142622
352	502.9	88.1	OFF	23.6	55.9	143742
346.8	489.2	85.6	OFF	23.6	55.9	144882
352	469.2	84	OFF	23.6	55.9	146011
352	462.7	82.4	OFF	23.6	55.9	147151
346.8	456.3	82.4	OFF	23.6	55.9	148270
352	456.3	81.6	OFF	23.6	55.9	149409
352	449.9	80.8	OFF	23.6	55.9	150529
352	443.7	80	OFF	23.6	55.9	151669
352	431.4	78.5	OFF	23.6	55.9	152794
346.8	425.3	77	OFF	23.6	55.9	153940
346.8	413.5	76.3	OFF	23.6	55.9	155059
352	407.7	74.9	OFF	23.6	55.9	156199
346.8	402	74.2	OFF	23.6	55.9	157319
352	396.3	73.5	OFF	23.7	55.9	158458
346.8	390.7	72.8	OFF	23.7	55.9	159578
352	385.2	72.1	OFF	23.6	55.9	160718
346.8	385.2	71.4	OFF	23.6	55.9	161838
346.8	379.7	70.7	OFF	23.6	55.9	162978
346.8	379.7	71.4	OFF	23.6	55.9	164098
346.8	379.7	70.7	OFF	23.7	55.8	165238
346.8	374.3	70	OFF	23.7	55.8	166358
352	374.3	70	OFF	23.6	55.9	167499
352	369	68.7	OFF	23.6	55.9	168620
346.8	363.8	68.7	OFF	23.6	55.9	169760
352	363.8	68.7	OFF	23.6	55.9	170880
346.8	358.6	67.4	OFF	23.6	55.9	172019
346.8	358.6	66.8	OFF	23.6	55.9	173138
346.8	358.6	66.8	OFF	23.6	55.9	174277
346.8	353.5	66.2	OFF	23.6	55.9	175396
346.8	348.4	66.2	OFF	23.6	55.9	176535
346.8	348.4	65.5	OFF	23.6	55.9	177655
346.8	348.4	65.5	OFF	23.6	55.9	178795
352	343.4	64.9	OFF	23.6	55.9	179914
352	343.4	64.9	OFF	23.6	55.9	181054
346.8	343.4	64.3	OFF	23.6	55.9	182174
346.8	338.5	64.3	OFF	23.6	55.9	183320
346.8	338.5	64.3	OFF	23.6	55.9	184445
346.8	338.5	63.7	OFF	23.6	55.9	185585
346.8	338.5	64.3	OFF	23.6	55.9	186704
346.8	338.5	63.7	OFF	23.6	55.9	187844
346.8	338.5	63.7	OFF	23.6	55.9	188964
346.8	333.6	63.7	OFF	23.6	55.9	190104
346.8	333.6	63.1	OFF	23.6	55.9	191223
346.8	328.8	62.5	OFF	23.7	55.9	192362
346.8	328.8	62.5	OFF	23.7	55.9	193482
341.7	324	61.9	OFF	23.6	55.9	194621
346.8	324	61.3	OFF	23.6	55.9	195741
346.8	324	61.3	OFF	23.6	55.9	196881
346.8	324	61.3	OFF	23.6	55.9	198001
346.8	324	61.3	OFF	23.6	55.9	199142
346.8	324	61.9	OFF	23.6	55.9	200261
346.8	324	61.3	OFF	23.7	55.9	201402
346.8	324	61.3	OFF	23.7	55.9	202521
346.8	319.4	61.3	OFF	23.6	55.9	203662
346.8	319.4	61.3	OFF	23.6	55.9	204783
346.8	319.4	60.2	OFF	23.6	55.9	205922
346.8	314.7	60.2	OFF	23.6	55.9	207041
Test run 2						
346.8	188.3	38.4	OFF	23.2	52.4	15726
346.8	194.2	38.8	OFF	23.2	52.4	16844
341.7	191.2	38.8	OFF	23.4	55.8	17983
341.7	191.2	38.8	OFF	23.4	55.8	19101
341.7	191.2	38.8	OFF	23.4	55.9	20240
341.7	191.2	39.2	OFF	23.4	55.9	21358
341.7	191.2	38.8	OFF	23.4	56	22497
341.7	188.3	38.4	OFF	23.4	56	23615
341.7	188.3	38.4	OFF	23.5	56	24755
341.7	188.3	38.4	OFF	23.5	56	25873
341.7	188.3	38.8	OFF	23.5	56.1	27012
341.7	188.3	38.8	OFF	23.5	56.1	28130
341.7	188.3	38.8	OFF	23.5	56.1	29268
341.7	191.2	38.8	OFF	23.5	56.1	30391
341.7	188.3	38.4	OFF	23.5	56.1	31535
341.7	188.3	38.4	OFF	23.5	56.1	32654
341.7	188.3	38.8	OFF	23.5	56.1	33792
341.7	188.3	38.8	OFF	23.5	56.1	34910
341.7	188.3	38.4	OFF	23.5	56.1	36049
341.7	191.2	38.8	OFF	23.5	56.1	37166
341.7	188.3	38.8	OFF	23.5	56.1	38305
336.6	191.2	38.8	OFF	23.5	56.1	39423
336.6	191.2	38.8	OFF	23.5	56.2	40562
341.7	191.2	38.8	OFF	23.5	56.2	41678
336.6	191.2	38.8	OFF	23.5	56.1	42816
341.7	188.3	38.8	OFF	23.5	56.1	43933
341.7	191.2	38.8	OFF	23.5	56.1	45072
341.7	191.2	38.8	OFF	23.5	56.1	46190
336.6	191.2	38.8	OFF	23.5	56.1	47328
336.6	188.3	38.8	OFF	23.5	56.1	48444
336.6	188.3	38.8	OFF	23.5	56.1	49583
336.6	191.2	38.8	OFF	23.5	56.1	50700
336.6	191.2	38.8	OFF	23.6	56	51840
336.6	191.2	38.8	OFF	23.6	56	52958
341.7	191.2	38.8	OFF	23.6	56	54098
336.6	191.2	38.8	OFF	23.6	56	55216
336.6	191.2	38.8	OFF	23.6	56	56356
341.7	191.2	38.8	OFF	23.6	56	57474
336.6	191.2	38.8	OFF	23.6	56	58614
341.7	191.2	38.8	OFF	23.6	56	59732
336.6	188.3	38.8	OFF	23.6	56	60877
336.6	188.3	38.4	OFF	23.6	56	61999
336.6	188.3	38.4	OFF	23.6	56	63138
336.6	188.3	38.4	OFF	23.6	56	64256
336.6	188.3	38.4	OFF	23.6	56	65395
336.6	188.3	38.4	OFF	23.6	56	66513
336.6	188.3	38.4	OFF	23.6	56	67653
341.7	188.3	38.4	OFF	23.6	56	68771
373.2	188.3	38.4	OFF	23.6	56	69911
384.1	188.3	38.8	OFF	23.6	56	71029
384.1	188.3	38.4	OFF	23.6	56	72168
378.6	230.1	50.7	OFF	23.6	56	73286
373.2	413.5	79.3	OFF	23.7	55.9	74424
373.2	1062.1	156.1	ON	23.7	55.9	75544
430.1	1745.7	219.5	ON	23.6	55.9	76685
430.1	2269.7	261.5	ON	23.6	55.9	77805
430.1	2475.9	261.5	ON	23.6	55.9	78945
424.2	3017.8	296.5	ON	23.6	55.9	80064
424.2	3093.2	287.3	ON	23.6	55.9	81203
448.3	2767.8	253.4	ON	23.6	55.9	82322
448.3	2298.1	221.7	ON	23.6	55.9	83462
442.2	1906	194.3	ON	23.6	55.9	84582
442.2	1702.4	183	ON	23.6	55.9	85722
442.2	1681.1	186.7	ON	23.6	55.9	86842
442.2	1790.2	190.5	ON	23.7	55.9	87982
442.2	1639.2	177.6	ON	23.7	55.9	89102
442.2	1500.5	165.6	ON	23.6	55.9	90242
436.1	1355.5	154.5	ON	23.6	55.9	91367
436.1	1288	150	ON	23.6	55.9	92512
436.1	1239.5	147.1	ON	23.6	55.9	93631
436.1	1192.7	142.8	ON	23.6	55.9	94771
436.1	1118.4	138.7	ON	23.6	55.9	95891
436.1	1048.4	132.1	ON	23.6	55.9	97031
436.1	969.8	127	OFF	23.6	55.9	98150
373.2	785.9	106.7	OFF	23.6	55.9	99289
373.2	745.2	102.7	OFF	23.6	55.9	100408
367.8	706.5	98.8	OFF	23.6	55.9	101547
367.8	669.6	95.1	OFF	23.6	55.9	102667
373.2	634.4	92.4	OFF	23.6	55.9	103806
367.8	601	88.9	OFF	23.6	55.9	104926
367.8	576.9	86.4	OFF	23.6	55.9	106067
367.8	546.2	84	OFF	23.6	55.9	107187
362.5	524.2	81.6	OFF	23.7	55.9	108327
362.5	502.9	79.3	OFF	23.7	55.9	109446
362.5	489.2	78.5	OFF	23.6	55.9	110585
362.5	475.8	76.3	OFF	23.6	55.9	111704
362.5	456.3	74.2	OFF	23.7	55.9	112845
362.5	437.5	72.1	OFF	23.7	55.9	113965
362.5	425.3	70.7	OFF	23.6	55.9	115105
362.5	407.7	70	OFF	23.6	55.9	116225
362.5	402	68.7	OFF	23.7	55.9	117364
362.5	390.7	67.4	OFF	23.7	55.9	118483
362.5	379.7	66.8	OFF	23.6	55.9	119623
362.5	374.3	65.5	OFF	23.6	55.9	120743
362.5	363.8	64.9	OFF	23.7	55.9	121888
362.5	353.5	63.7	OFF	23.7	55.9	123014
362.5	348.4	63.1	OFF	23.6	55.9	124153
362.5	343.4	61.9	OFF	23.6	55.9	125273
378.6	338.5	60.7	OFF	23.7	55.9	126414
367.8	328.8	60.2	OFF	23.7	55.9	127534
373.2	324	59	OFF	23.7	55.9	128674
373.2	319.4	59	OFF	23.7	55.9	129794
367.8	314.7	58.5	OFF	23.7	55.9	130934
367.8	310.2	57.9	OFF	23.7	55.9	132054
367.8	310.2	57.4	OFF	23.7	55.9	133194
367.8	310.2	57.4	OFF	23.7	55.9	134314
367.8	305.7	56.8	OFF	23.7	55.9	135454
367.8	301.2	56.8	OFF	23.7	55.9	136573
373.2	301.2	56.3	OFF	23.7	56	137714
367.8	296.8	55.8	OFF	23.7	56	138833
367.8	296.8	55.2	OFF	23.6	55.9	139973
367.8	292.5	55.2	OFF	23.6	55.9	141093
367.8	288.2	54.7	OFF	23.7	56	142233
367.8	283.9	53.7	OFF	23.7	56	143352
367.8	279.8	53.2	OFF	23.7	55.9	144491
367.8	279.8	53.2	OFF	23.7	55.9	145611
367.8	275.6	52.7	OFF	23.6	55.9	146750
367.8	275.6	52.7	OFF	23.6	55.9	147868
367.8	271.6	52.2	OFF	23.7	56	149008
367.8	271.6	51.7	OFF	23.7	56	150127
367.8	267.6	51.7	OFF	23.7	56	151267
367.8	267.6	51.2	OFF	23.7	56	152391
367.8	263.6	50.7	OFF	23.7	56	153537
367.8	263.6	50.7	OFF	23.7	56	154657
367.8	263.6	50.7	OFF	23.7	56	155795
373.2	263.6	50.7	OFF	23.7	56	156914
367.8	259.7	50.2	OFF	23.7	56	158054
367.8	259.7	50.2	OFF	23.7	56	159173
367.8	259.7	49.7	OFF	23.7	56	160314
367.8	255.8	49.3	OFF	23.7	56	161433
373.2	255.8	49.7	OFF	23.7	56	162573
367.8	255.8	48.8	OFF	23.7	56	163691
373.2	252	49.3	OFF	23.7	56	164832
367.8	252	48.3	OFF	23.7	56	165950
367.8	252	48.8	OFF	23.7	56	167090
367.8	252	48.3	OFF	23.7	56	168208
367.8	252	48.3	OFF	23.7	56	169349
367.8	252	48.3	OFF	23.7	56	170469
367.8	252	47.9	OFF	23.7	55.9	171608
362.5	248.2	47.9	OFF	23.7	55.9	172728
367.8	248.2	47.9	OFF	23.7	56	173867
367.8	244.5	47.4	OFF	23.7	56	174986
367.8	244.5	47.4	OFF	23.7	56	176126
373.2	244.5	47	OFF	23.7	56	177244
367.8	244.5	47	OFF	23.7	56	178384
367.8	240.8	47	OFF	23.7	56	179502
367.8	240.8	46.5	OFF	23.7	56	180642
367.8	240.8	46.5	OFF	23.7	56	181761
367.8	248.2	46.1	OFF	23.7	56	182905
367.8	240.8	46.1	OFF	23.7	56	184030
367.8	237.2	46.1	OFF	23.7	56	185170
367.8	237.2	46.1	OFF	23.7	56	186289
362.5	237.2	45.7	OFF	23.7	56	187429
362.5	233.7	45.2	OFF	23.7	56	188548
362.5	230.1	44.8	OFF	23.7	56	189688
362.5	230.1	44.8	OFF	23.7	56	190807
362.5	230.1	44.8	OFF	23.7	56	191948
362.5	226.6	44.4	OFF	23.7	56	193067
362.5	226.6	44.4	OFF	23.7	55.9	194207
362.5	226.6	44.4	OFF	23.7	55.9	195327
Test run 3						
357.2	206.7	40.7	OFF	23.4	52.6	18557
362.5	206.7	41.1	OFF	23.4	52.6	19675
357.2	206.7	41.1	OFF	23.5	55.9	20813
357.2	206.7	41.1	OFF	23.5	55.9	21932
357.2	206.7	41.1	OFF	23.6	55.8	23071
357.2	206.7	41.1	OFF	23.6	55.8	24189
357.2	206.7	41.1	OFF	23.6	55.8	25328
357.2	206.7	40.7	OFF	23.6	55.8	26446
357.2	206.7	40.7	OFF	23.6	55.8	27586
357.2	206.7	40.7	OFF	23.6	55.8	28704
357.2	203.5	40.7	OFF	23.6	55.9	29843
362.5	203.5	40.7	OFF	23.6	55.9	30967
357.2	203.5	40.7	OFF	23.6	55.9	32111
357.2	203.5	40.7	OFF	23.6	55.9	33229
357.2	206.7	40.7	OFF	23.6	55.9	34367
362.5	203.5	41.1	OFF	23.6	55.9	35485
357.2	206.7	40.7	OFF	23.6	55.9	36623
357.2	206.7	40.7	OFF	23.6	55.9	37741
362.5	203.5	41.1	OFF	23.6	55.9	38879
362.5	206.7	40.7	OFF	23.6	55.9	39997
357.2	206.7	41.1	OFF	23.6	55.9	41135
362.5	206.7	40.7	OFF	23.6	55.9	42253
357.2	206.7	40.7	OFF	23.6	55.8	43391
362.5	203.5	40.7	OFF	23.6	55.8	44509
357.2	206.7	40.7	OFF	23.6	55.9	45647
362.5	206.7	41.1	OFF	23.6	55.9	46765
357.2	206.7	40.7	OFF	23.6	55.9	47903
357.2	206.7	41.1	OFF	23.6	55.9	49020
357.2	206.7	41.1	OFF	23.7	55.8	50159
362.5	206.7	41.1	OFF	23.7	55.8	51277
357.2	209.9	41.5	OFF	23.7	55.9	52416
357.2	206.7	41.5	OFF	23.7	55.9	53534
362.5	206.7	41.1	OFF	23.7	55.9	54673
357.2	206.7	41.1	OFF	23.7	55.9	55792
357.2	206.7	41.5	OFF	23.7	55.9	56932
362.5	206.7	41.1	OFF	23.7	55.9	58051
357.2	206.7	41.1	OFF	23.7	55.9	59190
362.5	206.7	41.1	OFF	23.7	55.9	60309
357.2	203.5	40.3	OFF	23.7	55.9	61452
352	203.5	40.3	OFF	23.7	55.9	62576
352	200.4	39.9	OFF	23.7	55.9	63714
352	200.4	40.3	OFF	23.7	55.9	64833
352	200.4	40.3	OFF	23.7	55.9	65972
352	200.4	39.9	OFF	23.7	55.9	67091
352	200.4	39.9	OFF	23.7	55.9	68230
352	200.4	39.9	OFF	23.7	55.9	69349
352	200.4	39.9	OFF	23.7	55.8	70487
352	200.4	39.9	OFF	23.7	55.8	71605
352	200.4	39.9	OFF	23.7	55.8	72744
352	200.4	39.9	OFF	23.7	55.8	73863
352	200.4	39.9	OFF	23.7	55.9	75002
352	203.5	40.3	OFF	23.7	55.9	76121
357.2	203.5	40.7	OFF	23.7	55.9	77261
357.2	203.5	40.7	OFF	23.7	55.9	78380
448.3	206.7	40.7	OFF	23.7	55.8	79518
492.8	443.7	92.4	OFF	23.7	55.8	80637
512.8	5186.6	392.4	ON	23.7	55.8	81776
606.3	10378.1	681.7	ON	23.7	55.8	82897
621.7	12691.7	665.4	ON	23.8	55.7	84038
614	11766.4	604.9	ON	23.8	55.7	85159
598.7	10641.4	570.3	ON	23.7	55.8	86300
591.2	9390.9	513.8	ON	23.7	55.8	87419
576.3	7602.7	443.4	ON	23.7	55.8	88559
561.8	6086.9	379.7	ON	23.7	55.8	89679
554.6	4937.5	337	ON	23.7	55.7	90819
540.4	4156.7	299.7	ON	23.7	55.7	91944
533.4	3542.3	272.6	ON	23.7	55.8	93090
526.5	3131.6	253.4	ON	23.7	55.8	94209
519.6	3131.6	258.7	ON	23.7	55.8	95349
512.8	3170.5	261.5	ON	23.7	55.8	96468
506.1	3055.3	250.8	ON	23.7	55.8	97607
506.1	2700.2	235.8	ON	23.7	55.8	98726
499.4	2538.1	226.3	ON	23.7	55.8	99865
499.4	2356	221.7	ON	23.7	55.8	100984
499.4	2186.6	215	ON	23.7	55.8	102124
499.4	2054.4	212.9	ON	23.7	55.8	103245
492.8	1882.2	204.4	ON	23.7	55.8	104386
486.3	1723.9	192.4	ON	23.7	55.8	105507
479.8	1558.5	183	ON	23.7	55.8	106647
479.8	1519.6	179.3	ON	23.7	55.8	107767
479.8	1444.5	174	ON	23.7	55.8	108907
479.8	1338.3	165.6	ON	23.7	55.8	110029
506.1	1598.4	190.5	ON	23.6	55.8	111170
526.5	1177.5	150	ON	23.6	55.8	112290
479.8	1104.1	144.2	ON	23.7	55.9	113430
473.4	1048.4	140	ON	23.7	55.9	114551
473.4	1008.4	137.3	ON	23.7	55.9	115690
473.4	982.5	134.7	OFF	23.7	55.9	116810
400.9	796.4	115.3	OFF	23.7	55.9	117952
400.9	775.6	113.1	OFF	23.7	55.9	119073
395.3	755.2	110.9	OFF	23.7	55.9	120215
395.3	735.4	108.8	OFF	23.7	55.9	121335
389.7	716	106.7	OFF	23.7	55.9	122481
395.3	678.7	103.7	OFF	23.7	55.9	123607
389.7	651.8	100.7	OFF	23.7	55.9	124747
389.7	634.4	98.8	OFF	23.7	55.9	125867
389.7	617.5	96.9	OFF	23.7	55.9	127008
389.7	592.9	95.1	OFF	23.7	55.9	128128
384.1	584.9	93.3	OFF	23.7	55.9	129267
384.1	569.1	92.4	OFF	23.7	55.9	130387
384.1	553.8	89.8	OFF	23.8	55.8	131528
384.1	546.2	88.9	OFF	23.8	55.8	132647
378.6	524.2	87.2	OFF	23.7	55.9	133785
378.6	517	85.6	OFF	23.7	55.9	134905
378.6	509.9	84.8	OFF	23.8	55.8	136046
378.6	496	84	OFF	23.8	55.8	137165
378.6	489.2	83.2	OFF	23.7	55.9	138304
378.6	482.4	82.4	OFF	23.7	55.9	139424
378.6	482.4	82.4	OFF	23.8	55.8	140564
378.6	469.2	80.8	OFF	23.8	55.8	141683
378.6	462.7	80	OFF	23.8	55.8	142823
378.6	456.3	79.3	OFF	23.8	55.8	143942
378.6	449.9	79.3	OFF	23.8	55.8	145082
378.6	443.7	77.8	OFF	23.8	55.8	146201
378.6	437.5	76.3	OFF	23.8	55.7	147340
378.6	425.3	75.6	OFF	23.8	55.7	148460
378.6	419.4	74.9	OFF	23.7	55.8	149600
378.6	407.7	73.5	OFF	23.7	55.8	150720
378.6	402	72.8	OFF	23.7	55.8	151860
378.6	396.3	72.1	OFF	23.7	55.8	152985
378.6	385.2	71.4	OFF	23.8	55.7	154130
389.7	385.2	70.7	OFF	23.8	55.7	155248
384.1	379.7	70	OFF	23.7	55.8	156389
378.6	374.3	70	OFF	23.7	55.8	157509
378.6	369	68.7	OFF	23.7	55.9	158648
378.6	363.8	68.7	OFF	23.7	55.9	159768
378.6	353.5	67.4	OFF	23.8	55.7	160908
373.2	348.4	66.8	OFF	23.8	55.7	162028
373.2	343.4	65.5	OFF	23.7	55.8	163167
373.2	338.5	64.9	OFF	23.7	55.8	164286
373.2	333.6	64.3	OFF	23.8	55.7	165424
373.2	333.6	63.7	OFF	23.8	55.7	166543
373.2	324	63.1	OFF	23.7	55.8	167684
373.2	319.4	62.5	OFF	23.7	55.8	168803
373.2	314.7	61.9	OFF	23.8	55.7	169941
373.2	314.7	61.3	OFF	23.8	55.7	171061
373.2	310.2	61.3	OFF	23.8	55.7	172200
373.2	305.7	60.2	OFF	23.8	55.7	173320
373.2	305.7	59.6	OFF	23.7	55.9	174461
373.2	301.2	59	OFF	23.7	55.9	175581
367.8	296.8	59	OFF	23.8	55.7	176720
367.8	296.8	58.5	OFF	23.8	55.7	177840
367.8	292.5	57.9	OFF	23.8	55.8	178979
367.8	292.5	57.4	OFF	23.8	55.8	180098
367.8	288.2	57.4	OFF	23.8	55.8	181238
367.8	288.2	57.4	OFF	23.8	55.8	182357
367.8	283.9	56.8	OFF	23.7	55.9	183501
367.8	283.9	56.8	OFF	23.7	55.9	184625
367.8	283.9	56.3	OFF	23.8	55.8	185766
367.8	283.9	56.3	OFF	23.8	55.8	186885
373.2	279.8	56.8	OFF	23.8	55.7	188024
367.8	279.8	56.3	OFF	23.8	55.7	189144
367.8	279.8	55.8	OFF	23.8	55.7	190285
367.8	279.8	55.8	OFF	23.8	55.7	191405
367.8	279.8	55.8	OFF	23.7	55.8	192544
367.8	279.8	55.2	OFF	23.7	55.8	193663
367.8	275.6	54.7	OFF	23.7	55.8	194804
367.8	275.6	54.7	OFF	23.7	55.8	195924
367.8	271.6	54.7	OFF	23.7	55.8	197063
367.8	271.6	53.7	OFF	23.7	55.8	198184
367.8	267.6	53.7	OFF	23.7	55.8	199324
Test run 4						
357.2	216.5	43.1	OFF	23.2	52.5	19074
362.5	216.5	43.1	OFF	23.2	52.5	20192
357.2	216.5	43.5	OFF	23.5	55.9	21329
357.2	219.8	43.5	OFF	23.5	55.9	22447
357.2	219.8	43.1	OFF	23.5	56	23586
357.2	219.8	43.1	OFF	23.5	56	24704
357.2	219.8	43.5	OFF	23.5	56	25843
357.2	219.8	43.5	OFF	23.5	56	26961
357.2	219.8	43.5	OFF	23.5	56	28099
357.2	219.8	43.5	OFF	23.5	56	29217
357.2	219.8	43.5	OFF	23.5	56	30362
357.2	219.8	43.5	OFF	23.5	56	31486
357.2	219.8	43.5	OFF	23.5	56	32625
357.2	219.8	43.5	OFF	23.5	56	33743
362.5	223.2	43.9	OFF	23.6	55.9	34882
357.2	223.2	43.9	OFF	23.6	55.9	36000
362.5	223.2	43.9	OFF	23.5	56	37140
357.2	223.2	44.4	OFF	23.5	56	38258
357.2	223.2	44.4	OFF	23.6	55.9	39396
357.2	223.2	44.4	OFF	23.6	55.9	40515
362.5	223.2	43.9	OFF	23.6	55.9	41654
357.2	226.6	44.4	OFF	23.6	55.9	42772
357.2	223.2	43.9	OFF	23.6	55.9	43911
357.2	223.2	43.9	OFF	23.6	55.9	45030
357.2	219.8	43.9	OFF	23.6	55.9	46169
357.2	219.8	43.5	OFF	23.6	55.9	47286
357.2	219.8	43.1	OFF	23.6	55.9	48425
357.2	216.5	42.7	OFF	23.6	55.9	49543
357.2	216.5	43.1	OFF	23.6	55.9	50681
357.2	216.5	42.7	OFF	23.6	55.9	51799
357.2	216.5	42.7	OFF	23.6	55.9	52937
357.2	213.2	42.3	OFF	23.6	55.9	54055
357.2	213.2	42.3	OFF	23.6	55.9	55194
357.2	213.2	42.3	OFF	23.6	55.9	56313
357.2	209.9	42.3	OFF	23.6	55.9	57452
357.2	209.9	42.3	OFF	23.6	55.9	58570
357.2	209.9	41.9	OFF	23.6	55.9	59709
357.2	209.9	41.9	OFF	23.6	55.9	60833
352	209.9	41.9	OFF	23.6	56	61980
352	209.9	41.5	OFF	23.6	56	63099
352	206.7	41.9	OFF	23.6	56	64238
352	206.7	41.9	OFF	23.6	56	65355
352	209.9	41.5	OFF	23.6	56	66494
357.2	206.7	41.5	OFF	23.6	56	67612
352	206.7	41.5	OFF	23.6	56	68751
352	206.7	41.9	OFF	23.6	56	69868
352	209.9	41.5	OFF	23.6	55.9	71006
352	209.9	41.5	OFF	23.6	55.9	72125
352	206.7	41.5	OFF	23.6	56	73265
352	206.7	41.9	OFF	23.6	56	74382
357.2	209.9	41.5	OFF	23.6	56	75522
352	206.7	41.5	OFF	23.6	56	76640
352	206.7	41.5	OFF	23.6	55.9	77779
362.5	206.7	41.5	OFF	23.6	55.9	78898
389.7	2054.4	202.3	ON	23.7	55.9	80038
460.7	10249	583.9	ON	23.7	55.9	81159
460.7	15162	715.6	ON	23.7	55.9	82300
460.7	15554.5	751.6	ON	23.7	55.9	83421
460.7	15754.8	742.4	ON	23.7	55.9	84560
460.7	15554.5	742.4	ON	23.7	55.9	85682
467	14969.7	698.4	ON	23.7	55.9	86823
460.7	11474	604.9	ON	23.7	55.9	87944
460.7	9046.3	531.9	ON	23.7	55.9	89085
454.5	7416.8	485.3	ON	23.7	55.9	90205
454.5	6801.8	458.6	ON	23.7	55.9	91351
454.5	5515.7	414.7	ON	23.7	55.9	92475
454.5	4758.7	392.4	ON	23.7	55.9	93615
454.5	4208.1	363.5	ON	23.7	55.9	94735
448.3	3586.2	326.3	ON	23.7	55.8	95875
448.3	3093.2	293.4	ON	23.7	55.8	96995
448.3	2802.2	281.3	ON	23.7	55.9	98134
442.2	2667	269.8	ON	23.7	55.9	99254
442.2	2569.7	261.5	ON	23.7	55.9	100393
442.2	2538.1	258.7	ON	23.7	55.9	101514
436.1	2326.9	245.7	ON	23.7	55.9	102656
436.1	2186.6	240.7	ON	23.7	55.9	103777
436.1	2028.9	226.3	ON	23.7	55.9	104919
436.1	1858.8	215	ON	23.7	55.9	106040
436.1	1790.2	206.5	ON	23.7	55.9	107180
436.1	1639.2	200.3	ON	23.7	55.9	108301
436.1	1639.2	198.3	ON	23.7	55.9	109442
436.1	1681.1	200.3	ON	23.7	55.9	110563
436.1	1639.2	198.3	ON	23.6	55.9	111705
430.1	1598.4	200.3	ON	23.6	55.9	112826
430.1	1598.4	202.3	ON	23.7	55.9	113966
430.1	1538.9	194.3	ON	23.7	55.9	115087
430.1	1408.2	183	ON	23.7	55.9	116229
430.1	1338.3	177.6	ON	23.7	55.9	117350
430.1	1239.5	168.9	ON	23.7	55.9	118490
430.1	1162.5	160.7	ON	23.7	55.9	119610
430.1	1132.9	159.2	ON	23.7	55.9	120752
430.1	1132.9	157.6	ON	23.7	55.9	121877
430.1	1118.4	153	ON	23.7	55.9	123023
430.1	1075.9	148.5	ON	23.7	55.9	124143
430.1	1021.6	144.2	ON	23.7	55.8	125285
430.1	969.8	137.3	OFF	23.7	55.8	126405
362.5	775.6	115.3	OFF	23.7	55.9	127545
362.5	735.4	112	OFF	23.7	55.9	128665
362.5	716	109.8	OFF	23.7	55.9	129807
362.5	687.8	106.7	OFF	23.7	55.9	130928
362.5	660.6	104.7	OFF	23.7	55.9	132068
362.5	625.9	102.7	OFF	23.7	55.9	133190
362.5	617.5	100.7	OFF	23.7	55.9	134331
362.5	592.9	97.8	OFF	23.7	55.9	135451
362.5	576.9	96	OFF	23.7	55.9	136590
362.5	553.8	93.3	OFF	23.7	55.9	137710
362.5	538.8	91.5	OFF	23.7	55.9	138851
362.5	524.2	89.8	OFF	23.7	55.9	139970
362.5	517	88.9	OFF	23.7	55.9	141109
357.2	502.9	87.2	OFF	23.7	55.9	142229
362.5	489.2	85.6	OFF	23.7	55.9	143369
362.5	482.4	84	OFF	23.7	55.9	144489
362.5	469.2	83.2	OFF	23.7	55.9	145628
362.5	462.7	81.6	OFF	23.7	55.9	146748
362.5	456.3	81.6	OFF	23.7	55.9	147889
357.2	449.9	80	OFF	23.7	55.9	149009
357.2	443.7	79.3	OFF	23.7	56	150148
357.2	431.4	77.8	OFF	23.7	56	151267
357.2	425.3	77	OFF	23.7	56	152413
357.2	419.4	76.3	OFF	23.7	56	153537
357.2	413.5	74.9	OFF	23.7	56	154677
357.2	407.7	74.2	OFF	23.7	56	155796
357.2	396.3	72.8	OFF	23.7	56	156937
357.2	396.3	72.8	OFF	23.7	56	158056
357.2	390.7	72.1	OFF	23.7	56	159196
357.2	385.2	70.7	OFF	23.7	56	160315
352	379.7	70.7	OFF	23.7	56	161455
357.2	374.3	70	OFF	23.7	56	162574
357.2	369	69.4	OFF	23.7	56	163715
352	369	69.4	OFF	23.7	56	164834
357.2	363.8	68.7	OFF	23.7	56	165974
357.2	363.8	69.4	OFF	23.7	56	167093
357.2	358.6	68.1	OFF	23.7	55.9	168233
352	363.8	68.1	OFF	23.7	55.9	169353
357.2	358.6	67.4	OFF	23.7	55.9	170492
357.2	353.5	66.8	OFF	23.7	55.9	171612
357.2	353.5	66.2	OFF	23.8	55.8	172753
357.2	348.4	66.2	OFF	23.8	55.8	173872
352	343.4	65.5	OFF	23.7	55.9	175011
352	343.4	64.9	OFF	23.7	55.9	176131
357.2	338.5	64.3	OFF	23.7	55.9	177270
352	333.6	63.7	OFF	23.7	55.9	178390
357.2	333.6	63.7	OFF	23.7	55.9	179529
357.2	328.8	63.1	OFF	23.7	55.9	180648
357.2	324	62.5	OFF	23.7	55.9	181789
352	324	62.5	OFF	23.7	55.9	182914
352	319.4	61.3	OFF	23.8	55.8	184059
357.2	314.7	61.3	OFF	23.8	55.8	185178
357.2	310.2	60.7	OFF	23.8	55.8	186318
357.2	310.2	60.7	OFF	23.8	55.8	187436
357.2	305.7	60.2	OFF	23.7	55.9	188576
357.2	305.7	59.6	OFF	23.7	55.9	189695
352	301.2	59	OFF	23.7	55.9	190836
357.2	301.2	59	OFF	23.7	55.9	191956
357.2	301.2	58.5	OFF	23.8	55.8	193094
357.2	296.8	58.5	OFF	23.8	55.8	194212
357.2	296.8	58.5	OFF	23.8	55.8	195353
357.2	292.5	57.9	OFF	23.8	55.8	196472
357.2	292.5	57.4	OFF	23.7	55.8	197612
352	292.5	57.4	OFF	23.7	55.8	198731
357.2	292.5	57.4	OFF	23.8	55.7	199870
357.2	292.5	56.8	OFF	23.8	55.7	200990
357.2	288.2	56.8	OFF	23.7	55.8	202131
467	305.7	61.3	OFF	23.7	55.8	203250
454.5	310.2	61.3	OFF	23.8	55.7	204389
448.3	310.2	61.3	OFF	23.8	55.7	205507
442.2	310.2	61.3	OFF	23.8	55.7	206647
357.2	310.2	60.7	OFF	23.8	55.7	207767
Test run 5						
400.9	185.3	38.4	OFF	23.4	52.5	17258
400.9	185.3	38.8	OFF	23.4	52.5	18375
400.9	185.3	38.4	OFF	23.6	55.9	19514
400.9	182.4	38.4	OFF	23.6	55.9	20632
400.9	182.4	38.4	OFF	23.6	55.9	21771
400.9	182.4	38.4	OFF	23.6	55.9	22889
400.9	182.4	38.1	OFF	23.6	56	24028
400.9	182.4	38.1	OFF	23.6	56	25146
395.3	182.4	38.1	OFF	23.6	56	26286
395.3	182.4	38.1	OFF	23.6	56	27404
400.9	182.4	38.1	OFF	23.6	56	28544
400.9	179.6	37.7	OFF	23.6	56	29662
400.9	179.6	37.7	OFF	23.6	56	30808
395.3	179.6	37.7	OFF	23.6	56	31931
400.9	179.6	37.7	OFF	23.7	56	33071
400.9	179.6	37.7	OFF	23.7	56	34189
400.9	179.6	37.7	OFF	23.7	56	35327
395.3	179.6	37.7	OFF	23.7	56	36445
400.9	179.6	37.7	OFF	23.7	56	37585
395.3	179.6	37.7	OFF	23.7	56	38702
395.3	179.6	37.7	OFF	23.7	56	39840
395.3	179.6	37.7	OFF	23.7	56	40957
400.9	179.6	37.7	OFF	23.7	56	42095
395.3	179.6	37.7	OFF	23.7	56	43212
400.9	182.4	37.7	OFF	23.7	56	44350
400.9	182.4	38.1	OFF	23.7	56	45468
400.9	182.4	38.1	OFF	23.7	56	46607
395.3	182.4	38.1	OFF	23.7	56	47725
395.3	182.4	38.1	OFF	23.7	56.1	48865
395.3	185.3	38.4	OFF	23.7	56.1	49983
395.3	185.3	38.4	OFF	23.8	55.9	51123
400.9	188.3	38.8	OFF	23.8	55.9	52241
395.3	188.3	38.8	OFF	23.8	55.9	53380
395.3	188.3	38.8	OFF	23.8	55.9	54499
395.3	188.3	39.2	OFF	23.7	56	55637
395.3	191.2	39.2	OFF	23.7	56	56754
395.3	191.2	39.2	OFF	23.8	55.9	57892
400.9	191.2	39.2	OFF	23.8	55.9	59011
400.9	191.2	39.6	OFF	23.8	55.9	60150
395.3	191.2	39.6	OFF	23.8	55.9	61272
400.9	191.2	39.6	OFF	23.8	55.9	62418
400.9	191.2	39.6	OFF	23.8	55.9	63536
400.9	191.2	39.6	OFF	23.8	55.8	64676
400.9	191.2	39.6	OFF	23.8	55.8	65794
400.9	194.2	39.6	OFF	23.8	55.9	66933
395.3	194.2	39.6	OFF	23.8	55.9	68051
395.3	194.2	39.6	OFF	23.8	55.8	69190
400.9	194.2	39.6	OFF	23.8	55.8	70308
395.3	194.2	39.6	OFF	23.8	55.8	71446
395.3	194.2	39.6	OFF	23.8	55.8	72564
400.9	194.2	39.9	OFF	23.8	55.9	73702
400.9	194.2	39.9	OFF	23.8	55.9	74821
395.3	194.2	39.6	OFF	23.8	55.8	75959
430.1	194.2	39.6	OFF	23.8	55.8	77077
430.1	850.7	39.9	OFF	23.8	55.8	78215
424.2	2186.6	90.6	ON	23.8	55.8	79334
460.7	4312.9	253.4	ON	23.8	55.8	80475
454.5	5938.7	371.5	ON	23.8	55.8	81594
454.5	6972	433.6	ON	23.8	55.8	82735
454.5	7416.8	463.8	ON	23.8	55.8	83854
448.3	7058.7	448.4	ON	23.8	55.8	84994
448.3	6972	438.4	ON	23.8	55.8	86113
448.3	5938.7	383.9	ON	23.8	55.8	87253
448.3	5060.5	359.6	ON	23.8	55.8	88372
448.3	4758.7	359.6	ON	23.8	55.8	89512
448.3	5123.1	392.4	ON	23.8	55.8	90631
448.3	5448.2	401.1	ON	23.8	55.8	91776
454.5	5653.2	419.3	ON	23.8	55.8	92901
467	5584	419.3	ON	23.8	55.8	94042
473.4	5250.8	401.1	ON	23.8	55.8	95161
479.8	4643.1	355.7	ON	23.8	55.8	96301
473.4	4006.1	319.4	ON	23.8	55.8	97419
479.8	3498.9	293.4	ON	23.8	55.8	98559
479.8	3093.2	267	ON	23.8	55.8	99678
479.8	2733.8	253.4	ON	23.8	55.7	100816
467	2538.1	245.7	ON	23.8	55.7	101937
467	2385.4	231	ON	23.8	55.7	103077
467	2132.8	217.2	ON	23.8	55.7	104198
467	1906	200.3	ON	23.8	55.8	105340
467	1767.8	194.3	ON	23.8	55.8	106460
467	1767.8	198.3	ON	23.8	55.8	107602
467	1745.7	198.3	ON	23.8	55.8	108722
460.7	1723.9	196.3	ON	23.8	55.7	109863
460.7	1681.1	192.4	ON	23.8	55.7	110984
460.7	1618.7	188.6	ON	23.8	55.8	112123
460.7	1538.9	183	ON	23.8	55.8	113242
460.7	1444.5	177.6	ON	23.8	55.8	114382
460.7	1372.9	170.6	ON	23.8	55.8	115501
454.5	1271.7	162.3	ON	23.8	55.8	116643
454.5	1177.5	154.5	ON	23.8	55.8	117763
460.7	1089.9	145.7	ON	23.8	55.7	118905
454.5	1021.6	140	ON	23.8	55.7	120026
454.5	969.8	134.7	OFF	23.8	55.7	121166
412.4	807	116.4	OFF	23.8	55.7	122293
418.3	796.4	117.5	OFF	23.8	55.8	123441
412.4	745.2	110.9	OFF	23.8	55.8	124561
412.4	716	106.7	OFF	23.8	55.8	125701
412.4	687.8	104.7	OFF	23.8	55.8	126821
412.4	669.6	103.7	OFF	23.8	55.8	127963
412.4	651.8	100.7	OFF	23.8	55.8	129083
412.4	617.5	97.8	OFF	23.8	55.8	130222
412.4	601	96	OFF	23.8	55.8	131341
412.4	584.9	94.2	OFF	23.8	55.8	132481
406.7	561.4	93.3	OFF	23.8	55.8	133600
412.4	553.8	91.5	OFF	23.7	55.9	134740
406.7	546.2	89.8	OFF	23.7	55.9	135860
406.7	538.8	88.9	OFF	23.8	55.8	137000
412.4	531.4	88.1	OFF	23.8	55.8	138119
406.7	509.9	86.4	OFF	23.8	55.8	139258
442.2	538.8	91.5	OFF	23.8	55.8	140377
436.1	524.2	89.8	OFF	23.8	55.8	141516
436.1	517	88.9	OFF	23.8	55.8	142635
430.1	509.9	88.1	OFF	23.8	55.7	143774
430.1	496	86.4	OFF	23.8	55.7	144894
430.1	489.2	84.8	OFF	23.8	55.9	146035
430.1	475.8	83.2	OFF	23.8	55.9	147155
430.1	462.7	82.4	OFF	23.8	55.9	148295
430.1	449.9	80	OFF	23.8	55.9	149415
436.1	431.4	78.5	OFF	23.8	55.9	150554
430.1	419.4	76.3	OFF	23.8	55.9	151674
430.1	402	74.9	OFF	23.8	55.9	152820
424.2	390.7	72.8	OFF	23.8	55.9	153945
424.2	379.7	72.1	OFF	23.8	55.8	155084
418.3	369	70.7	OFF	23.8	55.8	156202
424.2	363.8	69.4	OFF	23.8	55.8	157343
424.2	353.5	68.1	OFF	23.8	55.8	158461
424.2	348.4	67.4	OFF	23.8	55.8	159599
424.2	338.5	66.2	OFF	23.8	55.8	160718
424.2	333.6	65.5	OFF	23.8	55.8	161856
424.2	324	64.3	OFF	23.8	55.8	162975
430.1	324	64.3	OFF	23.7	55.9	164116
424.2	319.4	63.1	OFF	23.7	55.9	165236
430.1	314.7	63.1	OFF	23.8	55.8	166375
424.2	310.2	61.9	OFF	23.8	55.8	167494
424.2	305.7	61.3	OFF	23.8	55.8	168632
430.1	301.2	60.7	OFF	23.8	55.8	169750
424.2	296.8	59.6	OFF	23.8	55.8	170891
424.2	292.5	59	OFF	23.8	55.8	172010
424.2	288.2	58.5	OFF	23.8	55.8	173151
424.2	283.9	57.4	OFF	23.8	55.8	174270
424.2	279.8	56.8	OFF	23.8	55.8	175409
424.2	275.6	56.3	OFF	23.8	55.8	176528
424.2	275.6	55.8	OFF	23.8	55.8	177667
418.3	271.6	55.2	OFF	23.8	55.8	178786
424.2	267.6	54.7	OFF	23.8	55.8	179927
424.2	263.6	54.2	OFF	23.8	55.8	181046
424.2	263.6	53.7	OFF	23.8	55.8	182184
424.2	259.7	53.2	OFF	23.8	55.8	183308
424.2	255.8	52.7	OFF	23.8	55.8	184453
424.2	252	52.2	OFF	23.8	55.8	185572
424.2	252	51.7	OFF	23.8	55.8	186711
418.3	248.2	51.2	OFF	23.8	55.8	187830
418.3	244.5	51.2	OFF	23.8	55.7	188971
418.3	240.8	50.2	OFF	23.8	55.7	190091
418.3	240.8	50.2	OFF	23.8	55.8	191230
418.3	240.8	49.7	OFF	23.8	55.8	192349
418.3	237.2	49.3	OFF	23.8	55.8	193490
418.3	237.2	49.3	OFF	23.8	55.8	194609
418.3	233.7	48.8	OFF	23.8	55.8	195748
412.4	233.7	48.3	OFF	23.8	55.8	196867
412.4	230.1	48.3	OFF	23.7	55.9	198006
412.4	226.6	47.9	OFF	23.7	55.9	199126
412.4	230.1	47.9	OFF	23.8	55.8	200267
418.3	226.6	47.4	OFF	23.8	55.8	201386
412.4	226.6	47.4	OFF	23.8	55.8	202526
412.4	226.6	47.4	OFF	23.8	55.8	203645
418.3	223.2	47	OFF	23.7	55.9	204786
412.4	223.2	46.5	OFF	23.7	55.9	205906
412.4	223.2	46.5	OFF	23.8	55.8	207045
Test run 6						
406.7	185.3	38.4	OFF	23.6	53.3	16853
412.4	188.3	38.8	OFF	23.6	53.3	17971
412.4	185.3	38.8	OFF	23.8	55.8	19110
412.4	185.3	38.4	OFF	23.8	55.8	20228
412.4	185.3	38.4	OFF	23.8	55.8	21366
406.7	185.3	38.4	OFF	23.8	55.8	22484
412.4	182.4	38.4	OFF	23.8	55.8	23622
406.7	182.4	38.4	OFF	23.8	55.8	24740
412.4	185.3	38.4	OFF	23.8	55.8	25880
406.7	185.3	38.4	OFF	23.8	55.8	26998
412.4	185.3	38.8	OFF	23.8	55.8	28138
412.4	185.3	38.4	OFF	23.8	55.8	29256
412.4	185.3	38.8	OFF	23.8	55.8	30400
412.4	185.3	38.8	OFF	23.8	55.8	31524
406.7	185.3	38.8	OFF	23.8	55.8	32663
406.7	185.3	38.8	OFF	23.8	55.8	33780
412.4	185.3	38.8	OFF	23.8	55.8	34918
412.4	185.3	38.8	OFF	23.8	55.8	36036
412.4	185.3	38.4	OFF	23.8	55.8	37174
406.7	185.3	38.4	OFF	23.8	55.8	38292
406.7	185.3	38.8	OFF	23.8	55.8	39432
406.7	185.3	38.8	OFF	23.8	55.8	40550
406.7	185.3	38.8	OFF	23.8	55.8	41689
406.7	185.3	38.8	OFF	23.8	55.8	42807
406.7	185.3	38.8	OFF	23.8	55.8	43944
406.7	185.3	38.8	OFF	23.8	55.8	45062
406.7	185.3	38.8	OFF	23.8	55.8	46200
406.7	185.3	38.8	OFF	23.8	55.8	47317
406.7	185.3	38.8	OFF	23.8	55.8	48455
406.7	185.3	38.8	OFF	23.8	55.8	49573
406.7	185.3	38.4	OFF	23.8	55.8	50711
406.7	185.3	38.8	OFF	23.8	55.8	51828
406.7	185.3	38.4	OFF	23.8	55.7	52966
406.7	185.3	38.4	OFF	23.8	55.7	54085
400.9	185.3	38.4	OFF	23.8	55.7	55223
406.7	185.3	38.4	OFF	23.8	55.7	56341
406.7	182.4	38.4	OFF	23.8	55.8	57479
406.7	182.4	38.4	OFF	23.8	55.8	58597
400.9	185.3	38.4	OFF	23.8	55.8	59734
406.7	185.3	38.4	OFF	23.8	55.8	60857
406.7	185.3	38.4	OFF	23.8	55.7	62000
406.7	185.3	38.4	OFF	23.8	55.7	63119
400.9	185.3	38.4	OFF	23.8	55.7	64259
406.7	185.3	38.4	OFF	23.8	55.7	65378
406.7	185.3	38.4	OFF	23.8	55.7	66518
406.7	182.4	38.4	OFF	23.8	55.7	67636
400.9	185.3	38.4	OFF	23.8	55.7	68774
400.9	185.3	38.4	OFF	23.8	55.7	69892
406.7	182.4	38.1	OFF	23.8	55.7	71030
406.7	185.3	38.4	OFF	23.8	55.7	72148
442.2	185.3	38.1	OFF	23.8	55.7	73288
442.2	185.3	38.8	OFF	23.8	55.7	74407
436.1	374.3	79.3	OFF	23.8	55.8	75546
430.1	1578.3	228.6	ON	23.8	55.8	76666
486.3	3767.1	401.1	ON	23.8	55.7	77806
486.3	4998.6	443.4	ON	23.8	55.7	78925
473.4	5123.1	433.6	ON	23.8	55.7	80065
467	4817.6	405.6	ON	23.8	55.7	81184
467	4260.2	367.5	ON	23.8	55.7	82324
454.5	4105.9	359.6	ON	23.8	55.7	83443
454.5	3957.1	340.6	ON	23.8	55.7	84584
454.5	3586.2	309.4	ON	23.8	55.7	85704
460.7	3249.6	284.3	ON	23.8	55.7	86844
454.5	3170.5	293.4	ON	23.8	55.7	87963
460.7	3330.7	319.4	ON	23.8	55.7	89102
454.5	3289.9	319.4	ON	23.8	55.7	90220
448.3	2980.8	299.7	ON	23.8	55.7	91367
448.3	2506.8	258.7	ON	23.8	55.7	92492
448.3	2080.2	228.6	ON	23.8	55.7	93631
448.3	1906	219.5	ON	23.8	55.7	94749
448.3	1790.2	212.9	ON	23.8	55.7	95889
448.3	1767.8	210.7	ON	23.8	55.7	97008
448.3	1702.4	202.3	ON	23.8	55.7	98149
448.3	1519.6	188.6	ON	23.8	55.7	99268
448.3	1372.9	175.8	ON	23.8	55.7	100407
442.2	1208.2	162.3	ON	23.8	55.7	101527
448.3	1075.9	150	ON	23.8	55.7	102668
442.2	969.8	140	OFF	23.8	55.7	103788
406.7	817.7	122.2	OFF	23.8	55.7	104929
406.7	775.6	116.4	OFF	23.8	55.7	106050
406.7	745.2	113.1	OFF	23.8	55.7	107189
400.9	725.6	112	OFF	23.8	55.7	108308
400.9	697.1	108.8	OFF	23.8	55.8	109449
395.3	651.8	103.7	OFF	23.8	55.8	110569
395.3	617.5	100.7	OFF	23.8	55.8	111709
395.3	601	97.8	OFF	23.8	55.8	112829
389.7	584.9	96	OFF	23.8	55.8	113969
395.3	561.4	94.2	OFF	23.8	55.8	115088
395.3	546.2	92.4	OFF	23.8	55.8	116226
389.7	538.8	91.5	OFF	23.8	55.8	117345
389.7	517	88.9	OFF	23.8	55.8	118485
389.7	509.9	87.2	OFF	23.8	55.8	119604
389.7	509.9	86.4	OFF	23.8	55.8	120744
389.7	496	85.6	OFF	23.8	55.8	121867
389.7	482.4	84.8	OFF	23.8	55.8	123011
389.7	482.4	83.2	OFF	23.8	55.8	124130
389.7	482.4	83.2	OFF	23.8	55.8	125271
389.7	475.8	81.6	OFF	23.8	55.8	126390
389.7	469.2	81.6	OFF	23.8	55.8	127530
389.7	456.3	80	OFF	23.8	55.8	128648
384.1	456.3	79.3	OFF	23.8	55.8	129787
384.1	449.9	78.5	OFF	23.8	55.8	130906
384.1	437.5	77	OFF	23.8	55.9	132047
389.7	413.5	74.9	OFF	23.8	55.9	133166
384.1	396.3	72.8	OFF	23.8	55.9	134306
384.1	379.7	70.7	OFF	23.8	55.9	135426
384.1	369	69.4	OFF	23.8	55.9	136565
384.1	358.6	68.1	OFF	23.8	55.9	137684
384.1	348.4	66.8	OFF	23.8	55.9	138824
384.1	338.5	65.5	OFF	23.8	55.9	139944
389.7	333.6	64.3	OFF	23.8	55.9	141085
389.7	324	63.1	OFF	23.8	55.9	142205
389.7	319.4	62.5	OFF	23.8	55.9	143344
389.7	314.7	61.9	OFF	23.8	55.9	144464
389.7	310.2	60.7	OFF	23.8	55.9	145604
389.7	301.2	59.6	OFF	23.8	55.9	146724
384.1	296.8	59	OFF	23.8	56	147865
389.7	292.5	58.5	OFF	23.8	56	148984
389.7	288.2	57.9	OFF	23.8	55.9	150123
384.1	283.9	56.8	OFF	23.8	55.9	151243
384.1	279.8	55.8	OFF	23.8	55.9	152388
389.7	271.6	55.2	OFF	23.8	55.9	153513
389.7	271.6	54.7	OFF	23.8	55.9	154653
389.7	267.6	54.2	OFF	23.8	55.9	155772
384.1	263.6	53.7	OFF	23.8	55.9	156912
389.7	259.7	52.7	OFF	23.8	55.9	158032
384.1	255.8	52.7	OFF	23.8	55.9	159172
384.1	255.8	52.2	OFF	23.8	55.9	160291
384.1	252	51.7	OFF	23.8	55.9	161430
384.1	252	51.2	OFF	23.8	55.9	162551
378.6	248.2	50.7	OFF	23.8	55.9	163691
378.6	248.2	50.2	OFF	23.8	55.9	164811
378.6	244.5	49.7	OFF	23.8	55.9	165951
384.1	240.8	49.7	OFF	23.8	55.9	167071
378.6	240.8	48.8	OFF	23.8	55.9	168210
384.1	237.2	48.8	OFF	23.8	55.9	169330
384.1	237.2	48.8	OFF	23.8	55.9	170469
384.1	237.2	48.3	OFF	23.8	55.9	171589
384.1	237.2	48.3	OFF	23.8	55.8	172730
389.7	233.7	48.8	OFF	23.8	55.8	173849
389.7	237.2	48.8	OFF	23.8	55.9	174987
384.1	237.2	48.3	OFF	23.8	55.9	176107
389.7	240.8	48.8	OFF	23.8	55.9	177248
389.7	240.8	48.3	OFF	23.8	55.9	178367
389.7	240.8	48.3	OFF	23.8	55.9	179508
384.1	237.2	47.9	OFF	23.8	55.9	180628
384.1	237.2	47.9	OFF	23.8	55.9	181767
389.7	233.7	47.4	OFF	23.8	55.9	182892
384.1	233.7	47	OFF	23.8	55.9	184040
384.1	230.1	47	OFF	23.8	55.9	185160
384.1	230.1	47	OFF	23.8	56	186300
384.1	230.1	46.5	OFF	23.8	56	187419
384.1	230.1	46.5	OFF	23.8	56	188559
384.1	226.6	46.1	OFF	23.8	56	189678
378.6	226.6	46.1	OFF	23.8	56	190819
378.6	223.2	45.7	OFF	23.8	56	191938
Test run 7						
378.6	200.4	41.1	OFF	23.6	53.5	18855
378.6	200.4	41.1	OFF	23.6	53.5	19973
378.6	200.4	41.1	OFF	23.8	55.8	21113
378.6	200.4	40.7	OFF	23.8	55.8	22231
384.1	197.3	40.7	OFF	23.8	55.9	23371
384.1	197.3	40.7	OFF	23.8	55.9	24488
389.7	197.3	40.7	OFF	23.7	56	25626
384.1	197.3	40.7	OFF	23.7	56	26743
384.1	197.3	40.3	OFF	23.8	55.8	27882
384.1	197.3	40.7	OFF	23.8	55.8	29000
384.1	197.3	40.7	OFF	23.8	55.8	30144
384.1	197.3	40.3	OFF	23.8	55.8	31267
384.1	194.2	40.3	OFF	23.8	55.9	32405
384.1	194.2	40.3	OFF	23.8	55.9	33523
378.6	194.2	39.9	OFF	23.8	55.9	34663
378.6	194.2	39.9	OFF	23.8	55.9	35781
378.6	194.2	39.9	OFF	23.8	55.9	36919
378.6	194.2	39.9	OFF	23.8	55.9	38038
378.6	194.2	39.9	OFF	23.8	55.8	39176
378.6	194.2	39.6	OFF	23.8	55.8	40294
378.6	194.2	39.6	OFF	23.8	55.8	41434
378.6	191.2	39.6	OFF	23.8	55.8	42552
378.6	191.2	39.6	OFF	23.8	55.8	43691
378.6	191.2	39.6	OFF	23.8	55.8	44809
378.6	191.2	39.2	OFF	23.8	55.8	45947
378.6	188.3	39.2	OFF	23.8	55.8	47065
378.6	191.2	39.2	OFF	23.8	55.8	48203
378.6	188.3	39.2	OFF	23.8	55.8	49321
378.6	188.3	39.2	OFF	23.8	55.8	50461
378.6	188.3	38.8	OFF	23.8	55.8	51579
378.6	188.3	38.8	OFF	23.8	55.8	52718
378.6	188.3	38.8	OFF	23.8	55.8	53836
378.6	188.3	38.8	OFF	23.8	55.8	54974
373.2	185.3	38.8	OFF	23.8	55.8	56092
378.6	188.3	38.8	OFF	23.8	55.8	57232
378.6	185.3	38.8	OFF	23.8	55.8	58350
378.6	185.3	38.8	OFF	23.8	55.8	59488
378.6	185.3	38.4	OFF	23.8	55.8	60611
378.6	185.3	38.4	OFF	23.7	55.9	61755
378.6	185.3	38.1	OFF	23.7	55.9	62872
378.6	185.3	38.4	OFF	23.8	55.8	64011
378.6	185.3	38.1	OFF	23.8	55.8	65129
378.6	185.3	38.1	OFF	23.8	55.8	66268
378.6	182.4	38.1	OFF	23.8	55.8	67386
378.6	182.4	38.1	OFF	23.8	55.8	68523
378.6	185.3	38.1	OFF	23.8	55.8	69641
378.6	185.3	38.1	OFF	23.8	55.8	70781
378.6	185.3	38.1	OFF	23.8	55.8	71899
378.6	185.3	38.1	OFF	23.8	55.8	73036
378.6	185.3	38.1	OFF	23.8	55.8	74154
378.6	185.3	38.1	OFF	23.8	55.8	75292
378.6	182.4	37.7	OFF	23.8	55.8	76409
378.6	185.3	38.1	OFF	23.8	55.8	77548
418.3	188.3	37.7	OFF	23.8	55.8	78666
424.2	2298.1	256.1	ON	23.8	55.8	79806
479.8	10121.6	665.4	ON	23.8	55.8	80925
473.4	15754.8	862.3	ON	23.8	55.7	82066
460.7	17915.8	907.3	ON	23.8	55.7	83187
460.7	17236.1	873.3	ON	23.8	55.7	84327
454.5	15356.9	789.7	ON	23.8	55.7	85448
448.3	14226	770.4	ON	23.8	55.7	86589
454.5	13693.9	751.6	ON	23.8	55.7	87709
448.3	13182.7	733.3	ON	23.7	55.8	88849
448.3	11915.6	698.4	ON	23.7	55.8	89970
442.2	12066.7	690	ON	23.8	55.7	91117
442.2	12375.1	634.3	ON	23.8	55.7	92242
442.2	9628.1	570.3	ON	23.8	55.8	93381
442.2	7989.1	525.8	ON	23.8	55.8	94500
436.1	6972	490.8	ON	23.8	55.8	95640
436.1	6238.9	453.4	ON	23.8	55.8	96759
442.2	5315.8	401.1	ON	23.7	55.9	97900
436.1	4530.3	351.8	ON	23.7	55.9	99020
442.2	4055.7	337	ON	23.8	55.8	100159
436.1	4006.1	359.6	ON	23.8	55.8	101279
436.1	4156.7	371.5	ON	23.7	55.9	102419
436.1	4156.7	375.6	ON	23.7	55.9	103540
436.1	4105.9	371.5	ON	23.7	55.9	104682
430.1	4055.7	371.5	ON	23.7	55.9	105803
430.1	3675.5	329.8	ON	23.7	55.9	106944
430.1	3017.8	296.5	ON	23.7	55.9	108065
430.1	2802.2	284.3	ON	23.7	55.9	109205
430.1	2700.2	284.3	ON	23.7	55.9	110326
430.1	2506.8	272.6	ON	23.7	55.9	111467
430.1	2186.6	253.4	ON	23.7	55.9	112588
430.1	2003.7	238.2	ON	23.7	55.9	113730
430.1	1745.7	217.2	ON	23.7	55.9	114851
430.1	1519.6	196.3	ON	23.7	55.9	115990
424.2	1355.5	183	ON	23.7	55.9	117111
430.1	1223.7	170.6	ON	23.7	55.9	118252
430.1	1118.4	162.3	ON	23.7	55.9	119373
424.2	1048.4	156.1	ON	23.7	55.9	120515
424.2	982.5	151.5	OFF	23.7	55.9	121643
389.7	850.7	133.4	OFF	23.7	56	122788
389.7	828.6	132.1	OFF	23.7	56	123908
389.7	817.7	130.8	OFF	23.7	56	125050
389.7	850.7	133.4	OFF	23.7	56	126170
389.7	839.6	130.8	OFF	23.7	56	127312
389.7	785.9	125.8	OFF	23.7	56	128431
389.7	745.2	121	OFF	23.7	56	129572
389.7	706.5	116.4	OFF	23.7	56	130692
389.7	660.6	112	OFF	23.7	56	131832
384.1	625.9	108.8	OFF	23.7	56	132951
389.7	601	104.7	OFF	23.6	56	134094
389.7	584.9	103.7	OFF	23.6	56	135214
389.7	561.4	100.7	OFF	23.7	56	136355
389.7	531.4	96.9	OFF	23.7	56	137473
389.7	509.9	94.2	OFF	23.7	56	138612
384.1	502.9	92.4	OFF	23.7	56	139731
384.1	496	91.5	OFF	23.7	56	140870
384.1	482.4	88.9	OFF	23.7	56	141989
384.1	462.7	87.2	OFF	23.7	56	143128
384.1	462.7	87.2	OFF	23.7	56	144247
384.1	456.3	85.6	OFF	23.7	56	145387
384.1	443.7	83.2	OFF	23.7	56	146506
384.1	425.3	81.6	OFF	23.7	56	147646
384.1	419.4	79.3	OFF	23.7	56	148764
384.1	407.7	77.8	OFF	23.7	56.1	149903
384.1	396.3	76.3	OFF	23.7	56.1	151022
384.1	390.7	75.6	OFF	23.7	56.1	152165
384.1	379.7	74.2	OFF	23.7	56.1	153290
384.1	374.3	73.5	OFF	23.7	56.1	154430
384.1	374.3	72.8	OFF	23.7	56.1	155549
384.1	369	71.4	OFF	23.7	56.1	156690
384.1	363.8	70.7	OFF	23.7	56.1	157809
384.1	358.6	70	OFF	23.7	56.1	158949
384.1	353.5	69.4	OFF	23.7	56.1	160068
384.1	353.5	69.4	OFF	23.7	56.1	161207
378.6	348.4	68.7	OFF	23.7	56.1	162326
384.1	348.4	67.4	OFF	23.7	56.1	163466
384.1	343.4	67.4	OFF	23.7	56.1	164585
384.1	338.5	66.2	OFF	23.7	56.1	165725
384.1	333.6	65.5	OFF	23.7	56.1	166844
384.1	324	64.3	OFF	23.7	56	167985
384.1	319.4	63.7	OFF	23.7	56	169105
384.1	314.7	63.1	OFF	23.7	56	170245
384.1	310.2	62.5	OFF	23.7	56	171364
384.1	310.2	61.9	OFF	23.7	56	172503
384.1	301.2	60.7	OFF	23.7	56	173622
384.1	296.8	60.2	OFF	23.7	56	174761
389.7	292.5	61.9	OFF	23.7	56	175880
384.1	292.5	59	OFF	23.7	55.9	177021
384.1	288.2	58.5	OFF	23.7	55.9	178142
384.1	283.9	57.9	OFF	23.7	56	179282
384.1	279.8	57.4	OFF	23.7	56	180400
384.1	279.8	56.8	OFF	23.7	55.9	181541
384.1	275.6	56.3	OFF	23.7	55.9	182666
384.1	275.6	55.8	OFF	23.7	56	183811
384.1	271.6	55.2	OFF	23.7	56	184930
384.1	271.6	54.7	OFF	23.7	56	186070
384.1	267.6	54.2	OFF	23.7	56	187189
384.1	263.6	53.7	OFF	23.7	55.9	188329
384.1	263.6	53.7	OFF	23.7	55.9	189449
384.1	259.7	52.7	OFF	23.7	55.9	190589
384.1	259.7	52.7	OFF	23.7	55.9	191708
384.1	255.8	52.2	OFF	23.7	56	192848
384.1	255.8	51.7	OFF	23.7	56	193966
424.2	305.7	57.9	OFF	23.7	56	195106
384.1	252	51.7	OFF	23.7	56	196224
384.1	248.2	51.2	OFF	23.7	56	197364
384.1	248.2	50.7	OFF	23.7	56	198483
Test run 8						
357.2	188.3	38.8	OFF	23.5	53.2	17219
357.2	188.3	38.4	OFF	23.5	53.2	18337
357.2	188.3	38.8	OFF	23.7	56.1	19476
357.2	188.3	38.8	OFF	23.7	56.1	20594
357.2	191.2	38.8	OFF	23.7	56.1	21732
357.2	191.2	38.8	OFF	23.7	56.1	22850
357.2	194.2	38.8	OFF	23.7	56.1	23988
357.2	194.2	39.2	OFF	23.7	56.1	25106
352	197.3	39.6	OFF	23.7	56.1	26244
357.2	200.4	39.2	OFF	23.7	56.1	27361
357.2	203.5	39.2	OFF	23.7	56.1	28501
357.2	203.5	39.9	OFF	23.7	56.1	29619
357.2	200.4	39.9	OFF	23.8	56	30764
357.2	203.5	40.3	OFF	23.8	56	31888
357.2	206.7	40.7	OFF	23.7	56.2	33026
357.2	206.7	40.7	OFF	23.7	56.2	34145
357.2	206.7	40.7	OFF	23.7	56.2	35284
357.2	206.7	40.7	OFF	23.7	56.2	36402
357.2	206.7	40.3	OFF	23.7	56.2	37539
357.2	200.4	39.9	OFF	23.7	56.2	38657
357.2	200.4	39.6	OFF	23.7	56.2	39795
357.2	197.3	39.6	OFF	23.7	56.2	40912
352	194.2	39.2	OFF	23.8	56	42053
357.2	194.2	39.2	OFF	23.8	56	43171
357.2	191.2	38.8	OFF	23.7	56.1	44309
357.2	191.2	38.4	OFF	23.7	56.1	45427
357.2	188.3	38.4	OFF	23.7	56.1	46566
352	188.3	38.4	OFF	23.7	56.1	47684
357.2	188.3	38.4	OFF	23.7	56.1	48823
357.2	188.3	38.1	OFF	23.7	56.1	49941
352	188.3	38.1	OFF	23.7	56.2	51079
352	185.3	38.1	OFF	23.7	56.2	52197
352	185.3	38.1	OFF	23.7	56.2	53337
357.2	185.3	38.1	OFF	23.7	56.2	54455
357.2	185.3	38.1	OFF	23.7	56.1	55594
352	185.3	38.1	OFF	23.7	56.1	56712
357.2	188.3	38.1	OFF	23.7	56.1	57850
357.2	188.3	38.1	OFF	23.7	56.1	58968
357.2	188.3	38.1	OFF	23.8	56	60107
362.5	188.3	38.1	OFF	23.8	56	61230
357.2	188.3	38.1	OFF	23.7	56.2	62375
357.2	188.3	38.1	OFF	23.7	56.2	63493
357.2	188.3	38.4	OFF	23.7	56.1	64633
357.2	188.3	38.4	OFF	23.7	56.1	65751
357.2	191.2	38.4	OFF	23.7	56.1	66889
357.2	191.2	38.4	OFF	23.7	56.1	68007
357.2	191.2	38.8	OFF	23.7	56.1	69146
357.2	191.2	38.8	OFF	23.7	56.1	70264
357.2	191.2	38.8	OFF	23.7	56.1	71403
357.2	191.2	38.8	OFF	23.7	56.1	72521
357.2	191.2	38.8	OFF	23.8	56	73660
357.2	191.2	38.8	OFF	23.8	56	74778
357.2	191.2	38.8	OFF	23.7	56.1	75916
357.2	191.2	38.8	OFF	23.7	56.1	77034
406.7	191.2	38.8	OFF	23.8	56	78175
406.7	2003.7	226.3	ON	23.8	56	79294
454.5	6972	525.8	ON	23.8	55.9	80434
448.3	10378.1	673.5	ON	23.8	55.9	81554
448.3	11474	706.9	ON	23.8	55.9	82694
448.3	11049.5	673.5	ON	23.8	55.9	83815
454.5	10641.4	673.5	ON	23.7	56.1	84955
448.3	10508.9	673.5	ON	23.7	56.1	86075
442.2	10249	665.4	ON	23.7	56	87217
442.2	9508.7	612.1	ON	23.7	56	88336
442.2	8500.7	590.8	ON	23.7	56	89476
442.2	7989.1	563.7	ON	23.7	56	90595
436.1	6886.4	513.8	ON	23.7	56.1	91740
436.1	5515.7	428.7	ON	23.7	56.1	92865
430.1	4420.2	379.7	ON	23.7	56.1	94005
430.1	3767.1	337	ON	23.7	56.1	95124
418.3	3131.6	296.5	ON	23.7	56.1	96264
418.3	2767.8	278.4	ON	23.7	56.1	97383
418.3	2634.2	267	ON	23.7	56.1	98523
418.3	2475.9	258.7	ON	23.7	56.1	99643
418.3	2298.1	243.2	ON	23.7	56.1	100782
418.3	2028.9	224	ON	23.7	56.1	101902
418.3	1812.8	206.5	ON	23.7	56.1	103044
418.3	1598.4	190.5	ON	23.7	56.1	104164
412.4	1426.2	174	ON	23.7	56.1	105304
412.4	1271.7	164	ON	23.7	56.1	106423
412.4	1147.6	154.5	ON	23.7	56.1	107564
412.4	1048.4	145.7	ON	23.7	56.1	108684
412.4	982.5	138.7	OFF	23.7	56.1	109825
362.5	807	119.8	OFF	23.7	56.1	110945
362.5	755.2	114.2	OFF	23.7	56.1	112085
362.5	716	109.8	OFF	23.7	56.1	113205
362.5	678.7	106.7	OFF	23.7	56.1	114347
362.5	643.1	103.7	OFF	23.7	56.1	115466
362.5	625.9	101.7	OFF	23.7	56.1	116606
362.5	609.2	99.7	OFF	23.7	56.1	117725
357.2	576.9	96	OFF	23.7	56.1	118864
357.2	561.4	93.3	OFF	23.7	56.1	119983
362.5	538.8	90.6	OFF	23.7	56.1	121124
357.2	524.2	89.8	OFF	23.7	56.1	122247
357.2	524.2	88.1	OFF	23.7	56.1	123392
357.2	509.9	87.2	OFF	23.7	56.1	124511
357.2	502.9	86.4	OFF	23.7	56.1	125649
357.2	496	84.8	OFF	23.7	56.1	126768
357.2	489.2	83.2	OFF	23.7	56.1	127907
357.2	475.8	82.4	OFF	23.7	56.1	129026
357.2	462.7	81.6	OFF	23.7	56.1	130167
357.2	456.3	80	OFF	23.7	56.1	131286
362.5	449.9	80	OFF	23.7	56.1	132425
357.2	443.7	79.3	OFF	23.7	56.1	133544
357.2	437.5	77	OFF	23.7	56.1	134684
357.2	431.4	76.3	OFF	23.7	56.1	135803
357.2	419.4	74.9	OFF	23.7	56.2	136943
357.2	407.7	73.5	OFF	23.7	56.2	138062
352	402	72.8	OFF	23.7	56.2	139201
357.2	396.3	71.4	OFF	23.7	56.2	140320
357.2	390.7	70.7	OFF	23.7	56.2	141461
357.2	385.2	69.4	OFF	23.7	56.2	142580
357.2	374.3	68.7	OFF	23.7	56.2	143719
357.2	407.7	68.7	OFF	23.7	56.2	144838
357.2	369	68.1	OFF	23.7	56.2	145979
357.2	363.8	67.4	OFF	23.7	56.2	147098
357.2	353.5	66.2	OFF	23.7	56.2	148237
357.2	343.4	64.9	OFF	23.7	56.2	149356
357.2	333.6	63.7	OFF	23.7	56.3	150496
357.2	328.8	62.5	OFF	23.7	56.3	151615
357.2	319.4	61.9	OFF	23.7	56.1	152759
357.2	314.7	60.7	OFF	23.7	56.1	153884
357.2	310.2	60.2	OFF	23.7	56.2	155023
357.2	319.4	61.3	OFF	23.7	56.2	156142
352	319.4	60.7	OFF	23.7	56.2	157283
357.2	314.7	60.7	OFF	23.7	56.2	158402
357.2	310.2	60.2	OFF	23.7	56.1	159541
357.2	305.7	59	OFF	23.7	56.1	160660
357.2	301.2	58.5	OFF	23.7	56.2	161801
357.2	292.5	57.4	OFF	23.7	56.2	162920
357.2	292.5	56.8	OFF	23.7	56.1	164059
357.2	288.2	56.8	OFF	23.7	56.1	165178
357.2	283.9	55.8	OFF	23.7	56.2	166318
357.2	283.9	55.2	OFF	23.7	56.2	167437
357.2	279.8	55.2	OFF	23.7	56.2	168576
352	279.8	54.2	OFF	23.7	56.2	169695
357.2	275.6	54.2	OFF	23.7	56.2	170836
357.2	275.6	53.7	OFF	23.7	56.2	171955
357.2	271.6	53.7	OFF	23.8	56.1	173095
357.2	271.6	53.2	OFF	23.8	56.1	174214
357.2	271.6	53.2	OFF	23.7	56.3	175355
352	267.6	52.2	OFF	23.7	56.3	176474
357.2	267.6	52.2	OFF	23.7	56.3	177613
357.2	263.6	52.2	OFF	23.7	56.3	178731
357.2	263.6	51.2	OFF	23.7	56.3	179871
357.2	259.7	51.2	OFF	23.7	56.3	180990
357.2	259.7	50.7	OFF	23.7	56.3	182131
357.2	259.7	50.7	OFF	23.7	56.3	183255
357.2	259.7	50.7	OFF	23.7	56.3	184401
357.2	263.6	51.2	OFF	23.7	56.3	185520
357.2	271.6	52.7	OFF	23.7	56.2	186658
357.2	279.8	52.7	OFF	23.7	56.2	187777
357.2	275.6	52.2	OFF	23.7	56.3	188916
357.2	267.6	51.2	OFF	23.7	56.3	190034
357.2	263.6	50.7	OFF	23.7	56.3	191174
357.2	259.7	50.7	OFF	23.7	56.3	192293
352	255.8	49.7	OFF	23.8	56.2	193433
357.2	255.8	49.7	OFF	23.8	56.2	194551
352	252	49.3	OFF	23.8	56.2	195691
357.2	248.2	48.8	OFF	23.8	56.2	196810
Test run 9						
357.2	226.6	44.8	OFF	23.6	53.7	16538
357.2	226.6	44.4	OFF	23.6	53.7	17656
362.5	223.2	44.4	OFF	23.7	56.3	18794
362.5	223.2	43.9	OFF	23.7	56.3	19912
362.5	219.8	43.5	OFF	23.7	56.4	21050
357.2	219.8	43.5	OFF	23.7	56.4	22168
357.2	219.8	43.1	OFF	23.7	56.3	23307
357.2	216.5	43.1	OFF	23.7	56.3	24424
357.2	216.5	42.7	OFF	23.7	56.3	25563
362.5	213.2	42.7	OFF	23.7	56.3	26681
362.5	213.2	42.7	OFF	23.7	56.3	27821
362.5	213.2	42.3	OFF	23.7	56.3	28939
357.2	213.2	42.3	OFF	23.7	56.3	30079
362.5	209.9	42.3	OFF	23.7	56.3	31202
367.8	213.2	41.9	OFF	23.7	56.3	32346
362.5	213.2	42.3	OFF	23.7	56.3	33466
362.5	216.5	42.3	OFF	23.8	56.2	34605
357.2	216.5	42.7	OFF	23.8	56.2	35723
362.5	216.5	42.3	OFF	23.8	56.2	36861
362.5	213.2	42.3	OFF	23.8	56.2	37978
362.5	213.2	42.3	OFF	23.8	56.2	39117
362.5	213.2	42.3	OFF	23.8	56.2	40234
362.5	213.2	42.3	OFF	23.8	56.2	41373
362.5	213.2	42.3	OFF	23.8	56.2	42490
362.5	213.2	42.3	OFF	23.8	56.2	43629
362.5	213.2	42.3	OFF	23.8	56.2	44746
357.2	213.2	42.3	OFF	23.7	56.3	45884
362.5	213.2	42.3	OFF	23.7	56.3	47001
362.5	213.2	42.3	OFF	23.8	56.2	48140
362.5	213.2	42.3	OFF	23.8	56.2	49257
362.5	213.2	42.3	OFF	23.8	56.2	50397
362.5	213.2	42.3	OFF	23.8	56.2	51514
362.5	213.2	42.3	OFF	23.8	56.2	52654
367.8	213.2	42.3	OFF	23.8	56.2	53773
362.5	213.2	41.9	OFF	23.8	56.2	54913
362.5	209.9	41.9	OFF	23.8	56.2	56031
362.5	209.9	41.9	OFF	23.8	56.2	57172
367.8	209.9	41.9	OFF	23.8	56.2	58289
367.8	209.9	41.5	OFF	23.8	56.2	59430
367.8	209.9	41.5	OFF	23.8	56.2	60548
362.5	209.9	41.5	OFF	23.8	56.2	61694
362.5	209.9	41.5	OFF	23.8	56.2	62818
362.5	206.7	41.5	OFF	23.8	56.2	63959
362.5	209.9	41.5	OFF	23.8	56.2	65077
367.8	209.9	41.1	OFF	23.8	56.2	66217
362.5	206.7	41.5	OFF	23.8	56.2	67335
362.5	206.7	41.1	OFF	23.8	56.2	68476
367.8	206.7	41.1	OFF	23.8	56.2	69594
362.5	206.7	41.1	OFF	23.8	56.1	70733
362.5	206.7	41.1	OFF	23.8	56.1	71851
395.3	206.7	41.1	OFF	23.8	56.1	72990
400.9	755.2	147.1	OFF	23.8	56.1	74109
400.9	4998.6	448.4	ON	23.8	55.9	75248
448.3	11915.6	780	ON	23.8	55.9	76368
486.3	17459.5	943	ON	23.8	55.9	77509
479.8	18148.7	943	ON	23.8	55.9	78629
486.3	18148.7	943	ON	23.7	56	79769
479.8	15554.5	840.7	ON	23.7	56	80889
479.8	12219.9	681.7	ON	23.7	56.2	82030
479.8	9390.9	550.7	ON	23.7	56.2	83149
479.8	7890.7	508	ON	23.7	56.3	84290
473.4	7509.1	496.5	ON	23.7	56.3	85409
473.4	7509.1	490.8	ON	23.7	56.3	86549
467	6554.5	463.8	ON	23.7	56.3	87668
467	6886.4	525.8	ON	23.7	56.3	88807
467	7235.5	525.8	ON	23.7	56.3	89926
460.7	6718.3	496.5	ON	23.7	56.3	91065
460.7	5938.7	453.4	ON	23.7	56.3	92189
460.7	5448.2	424	ON	23.7	56.3	93334
460.7	4877.2	383.9	ON	23.7	56.3	94452
454.5	4312.9	355.7	ON	23.6	56.3	95590
454.5	3957.1	340.6	ON	23.6	56.3	96708
454.5	3767.1	329.8	ON	23.7	56.3	97847
454.5	3498.9	309.4	ON	23.7	56.3	98966
454.5	3131.6	281.3	ON	23.7	56.3	100105
454.5	2802.2	261.5	ON	23.7	56.3	101224
448.3	2601.7	250.8	ON	23.6	56.3	102363
454.5	2415.2	245.7	ON	23.6	56.3	103482
448.3	2241.7	233.4	ON	23.7	56.3	104622
448.3	1978.9	215	ON	23.7	56.3	105742
448.3	1858.8	208.6	ON	23.6	56.3	106882
448.3	1767.8	200.3	ON	23.6	56.3	108002
448.3	1681.1	196.3	ON	23.6	56.3	109142
448.3	1538.9	183	ON	23.6	56.3	110261
442.2	1390.4	170.6	ON	23.7	56.3	111401
448.3	1255.5	160.7	ON	23.7	56.3	112521
448.3	1162.5	154.5	ON	23.7	56.3	113661
448.3	1118.4	150	ON	23.7	56.3	114781
442.2	1062.1	144.2	ON	23.7	56.3	115921
442.2	1008.4	138.7	ON	23.7	56.3	117042
442.2	969.8	134.7	OFF	23.6	56.3	118182
395.3	807	116.4	OFF	23.6	56.3	119302
395.3	755.2	112	OFF	23.7	56.3	120442
395.3	735.4	110.9	OFF	23.7	56.3	121561
389.7	716	107.7	OFF	23.7	56.3	122708
389.7	687.8	104.7	OFF	23.7	56.3	123834
395.3	643.1	99.7	OFF	23.6	56.3	124974
389.7	601	96	OFF	23.6	56.3	126092
389.7	584.9	95.1	OFF	23.7	56.2	127231
389.7	569.1	93.3	OFF	23.7	56.2	128351
389.7	546.2	89.8	OFF	23.7	56.2	129490
389.7	524.2	87.2	OFF	23.7	56.2	130609
389.7	496	84.8	OFF	23.7	56.3	131748
389.7	475.8	83.2	OFF	23.7	56.3	132866
389.7	462.7	80.8	OFF	23.7	56.3	134005
389.7	449.9	79.3	OFF	23.7	56.3	135122
389.7	431.4	77	OFF	23.7	56.3	136263
389.7	419.4	74.9	OFF	23.7	56.3	137382
389.7	413.5	74.2	OFF	23.7	56.3	138523
389.7	402	72.8	OFF	23.7	56.3	139642
389.7	396.3	71.4	OFF	23.7	56.3	140781
389.7	385.2	70.7	OFF	23.7	56.3	141899
389.7	374.3	69.4	OFF	23.7	56.3	143038
389.7	385.2	69.4	OFF	23.7	56.3	144156
389.7	385.2	68.7	OFF	23.7	56.3	145295
384.1	425.3	73.5	OFF	23.7	56.3	146413
384.1	443.7	73.5	OFF	23.7	56.3	147553
384.1	437.5	74.2	OFF	23.7	56.3	148672
384.1	431.4	74.2	OFF	23.7	56.3	149811
389.7	413.5	72.1	OFF	23.7	56.3	150929
384.1	402	70.7	OFF	23.7	56.3	152067
389.7	385.2	69.4	OFF	23.7	56.3	153189
389.7	374.3	67.4	OFF	23.7	56.3	154335
389.7	369	66.8	OFF	23.7	56.3	155453
384.1	358.6	66.2	OFF	23.7	56.4	156593
389.7	353.5	64.9	OFF	23.7	56.4	157712
389.7	348.4	64.3	OFF	23.7	56.3	158853
384.1	343.4	63.7	OFF	23.7	56.3	159972
389.7	338.5	63.1	OFF	23.7	56.4	161113
389.7	333.6	61.9	OFF	23.7	56.4	162232
389.7	328.8	61.9	OFF	23.7	56.4	163373
389.7	328.8	60.7	OFF	23.7	56.4	164492
395.3	324	60.2	OFF	23.7	56.4	165633
395.3	314.7	59.6	OFF	23.7	56.4	166752
389.7	310.2	59	OFF	23.7	56.3	167893
389.7	310.2	58.5	OFF	23.7	56.3	169012
389.7	305.7	57.9	OFF	23.6	56.3	170152
389.7	305.7	56.8	OFF	23.6	56.3	171271
389.7	301.2	56.3	OFF	23.8	56.3	172411
389.7	296.8	56.3	OFF	23.8	56.3	173530
389.7	292.5	55.2	OFF	23.7	56.4	174670
389.7	288.2	54.7	OFF	23.7	56.4	175789
389.7	288.2	54.7	OFF	23.7	56.4	176929
384.1	283.9	53.7	OFF	23.7	56.4	178048
384.1	283.9	53.7	OFF	23.6	56.4	179188
384.1	283.9	53.2	OFF	23.6	56.4	180307
384.1	279.8	53.2	OFF	23.7	56.4	181447
384.1	275.6	52.7	OFF	23.7	56.4	182565
384.1	275.6	52.2	OFF	23.7	56.4	183711
384.1	271.6	51.7	OFF	23.7	56.4	184836
384.1	267.6	51.2	OFF	23.8	56.3	185977
384.1	267.6	51.2	OFF	23.8	56.3	187095
389.7	263.6	50.7	OFF	23.8	56.2	188236
384.1	259.7	50.7	OFF	23.8	56.2	189354
384.1	259.7	50.2	OFF	23.8	56.2	190500
384.1	255.8	49.7	OFF	23.8	56.2	191618
400.9	259.7	50.2	OFF	23.7	56.3	192758
384.1	259.7	49.7	OFF	23.7	56.3	193876
Test run 10						
384.1	223.2	43.5	OFF	23.6	53.5	20371
384.1	223.2	43.1	OFF	23.6	53.5	21489
389.7	219.8	43.1	OFF	23.7	56.2	22628
384.1	219.8	43.1	OFF	23.7	56.2	23745
384.1	223.2	43.1	OFF	23.7	56.3	24884
384.1	219.8	42.7	OFF	23.7	56.3	26001
389.7	216.5	42.7	OFF	23.7	56.3	27140
384.1	216.5	42.7	OFF	23.7	56.3	28258
378.6	216.5	42.3	OFF	23.7	56.3	29398
384.1	216.5	42.7	OFF	23.7	56.3	30521
378.6	213.2	42.3	OFF	23.8	56.1	31668
378.6	213.2	41.9	OFF	23.8	56.1	32785
378.6	213.2	42.3	OFF	23.7	56.2	33925
378.6	213.2	41.9	OFF	23.7	56.2	35044
378.6	213.2	42.3	OFF	23.7	56.2	36183
378.6	213.2	41.9	OFF	23.7	56.2	37300
378.6	213.2	41.9	OFF	23.8	56.1	38440
384.1	219.8	41.9	OFF	23.8	56.1	39558
384.1	213.2	41.9	OFF	23.8	56.1	40698
384.1	213.2	41.9	OFF	23.8	56.1	41816
384.1	213.2	41.9	OFF	23.8	56.1	42956
384.1	213.2	41.9	OFF	23.8	56.1	44073
384.1	213.2	41.9	OFF	23.8	56.1	45214
384.1	213.2	41.9	OFF	23.8	56.1	46332
378.6	209.9	41.9	OFF	23.8	56.1	47472
378.6	209.9	41.9	OFF	23.8	56.1	48589
378.6	209.9	41.5	OFF	23.8	56.1	49728
384.1	209.9	41.5	OFF	23.8	56.1	50846
384.1	213.2	41.5	OFF	23.8	56.1	51985
378.6	209.9	41.5	OFF	23.8	56.1	53104
378.6	213.2	41.5	OFF	23.8	56.1	54242
378.6	209.9	41.5	OFF	23.8	56.1	55360
378.6	213.2	41.5	OFF	23.8	56.1	56499
384.1	209.9	41.1	OFF	23.8	56.1	57617
384.1	209.9	41.5	OFF	23.8	56.1	58757
378.6	209.9	41.5	OFF	23.8	56.1	59875
378.6	209.9	41.5	OFF	23.8	56.1	61020
384.1	209.9	41.5	OFF	23.8	56.1	62143
384.1	209.9	41.5	OFF	23.8	56.1	63282
384.1	209.9	41.5	OFF	23.8	56.1	64400
378.6	209.9	41.5	OFF	23.8	56	65539
378.6	209.9	41.5	OFF	23.8	56	66657
378.6	209.9	41.5	OFF	23.8	56.1	67796
384.1	209.9	41.5	OFF	23.8	56.1	68914
378.6	209.9	41.5	OFF	23.8	56	70053
378.6	209.9	41.5	OFF	23.8	56	71171
378.6	209.9	41.1	OFF	23.8	56.1	72310
378.6	213.2	41.5	OFF	23.8	56.1	73428
378.6	213.2	41.5	OFF	23.8	56	74567
378.6	213.2	41.5	OFF	23.8	56	75685
384.1	213.2	41.9	OFF	23.8	56	76825
378.6	213.2	41.5	OFF	23.8	56	77943
378.6	213.2	41.5	OFF	23.8	56	79083
418.3	209.9	41.5	OFF	23.8	56	80201
424.2	213.2	47	OFF	23.8	56	81341
418.3	1906	231	ON	23.8	56	82460
467	4877.2	414.7	ON	23.8	56	83601
460.7	5381.6	438.4	ON	23.8	56	84720
460.7	5381.6	424	ON	23.8	56	85861
454.5	5060.5	405.6	ON	23.8	56	86980
454.5	5060.5	401.1	ON	23.8	56	88122
448.3	4817.6	392.4	ON	23.8	56	89241
448.3	4877.2	392.4	ON	23.8	56.2	90382
448.3	4877.2	392.4	ON	23.8	56.2	91506
442.2	4758.7	383.9	ON	23.8	56.2	92651
442.2	4643.1	379.7	ON	23.8	56.2	93769
436.1	4530.3	371.5	ON	23.8	56.3	94909
442.2	3861	322.8	ON	23.8	56.3	96028
442.2	3131.6	278.4	ON	23.8	56.3	97168
442.2	2634.2	253.4	ON	23.8	56.3	98286
442.2	2506.8	253.4	ON	23.8	56.3	99425
436.1	2445.4	248.2	ON	23.8	56.3	100544
436.1	2269.7	233.4	ON	23.8	56.3	101686
436.1	2054.4	215	ON	23.8	56.3	102805
436.1	1882.2	202.3	ON	23.8	56.2	103947
436.1	1812.8	194.3	ON	23.8	56.2	105067
430.1	1767.8	192.4	ON	23.8	56.2	106209
436.1	1702.4	184.8	ON	23.8	56.2	107329
430.1	1558.5	174	ON	23.8	56.2	108472
430.1	1481.6	168.9	ON	23.8	56.2	109592
430.1	1390.4	162.3	ON	23.8	56.2	110734
430.1	1304.6	154.5	ON	23.8	56.2	111853
430.1	1304.6	157.6	ON	23.8	56.1	112995
430.1	1271.7	156.1	ON	23.8	56.1	114116
430.1	1177.5	147.1	ON	23.8	56.2	115257
424.2	1075.9	138.7	ON	23.8	56.2	116377
430.1	1008.4	132.1	ON	23.8	56.2	117518
430.1	932.5	125.8	OFF	23.8	56.2	118638
378.6	775.6	108.8	OFF	23.8	56.2	119779
378.6	735.4	104.7	OFF	23.8	56.2	120899
378.6	706.5	101.7	OFF	23.8	56.1	122046
378.6	678.7	99.7	OFF	23.8	56.1	123170
378.6	651.8	97.8	OFF	23.8	56.1	124310
378.6	634.4	95.1	OFF	23.8	56.1	125429
378.6	601	92.4	OFF	23.8	56.1	126569
378.6	576.9	89.8	OFF	23.8	56.1	127688
378.6	553.8	87.2	OFF	23.8	56.1	128829
378.6	531.4	84.8	OFF	23.8	56.1	129948
378.6	509.9	83.2	OFF	23.8	56.1	131090
378.6	496	81.6	OFF	23.8	56.1	132209
378.6	482.4	80	OFF	23.8	56.1	133351
378.6	469.2	78.5	OFF	23.8	56.1	134470
373.2	456.3	76.3	OFF	23.8	56.1	135611
378.6	443.7	75.6	OFF	23.8	56.1	136730
378.6	431.4	74.2	OFF	23.8	56.1	137870
378.6	425.3	72.1	OFF	23.8	56.1	138989
373.2	413.5	70.7	OFF	23.8	56.1	140131
373.2	402	70	OFF	23.8	56.1	141249
378.6	396.3	68.7	OFF	23.8	56.1	142390
378.6	385.2	67.4	OFF	23.8	56.1	143509
373.2	374.3	66.2	OFF	23.8	56.1	144650
373.2	363.8	64.9	OFF	23.8	56.1	145769
373.2	358.6	64.3	OFF	23.8	56.1	146911
373.2	348.4	63.7	OFF	23.8	56.1	148029
373.2	343.4	63.1	OFF	23.8	56.1	149170
378.6	338.5	61.9	OFF	23.8	56.1	150289
373.2	333.6	61.9	OFF	23.8	56.1	151431
378.6	328.8	61.3	OFF	23.8	56.1	152555
378.6	324	60.7	OFF	23.8	56	153702
378.6	324	60.2	OFF	23.8	56	154821
373.2	314.7	59	OFF	23.8	56.1	155961
378.6	310.2	58.5	OFF	23.8	56.1	157080
373.2	305.7	57.9	OFF	23.8	56	158219
373.2	305.7	57.4	OFF	23.8	56	159337
373.2	301.2	56.8	OFF	23.8	56.1	160477
378.6	296.8	56.3	OFF	23.8	56.1	161596
378.6	292.5	55.8	OFF	23.8	56.1	162737
378.6	288.2	55.2	OFF	23.8	56.1	163856
378.6	288.2	54.7	OFF	23.8	56	164996
378.6	283.9	54.2	OFF	23.8	56	166115
373.2	279.8	53.7	OFF	23.8	56	167255
378.6	279.8	53.2	OFF	23.8	56	168373
378.6	275.6	53.2	OFF	23.8	56	169514
373.2	271.6	52.7	OFF	23.8	56	170632
373.2	271.6	52.2	OFF	23.8	56	171773
373.2	267.6	52.2	OFF	23.8	56	172891
373.2	267.6	51.7	OFF	23.9	56	174031
373.2	263.6	51.2	OFF	23.9	56	175150
373.2	263.6	50.7	OFF	23.8	56	176291
373.2	259.7	50.7	OFF	23.8	56	177409
373.2	259.7	50.2	OFF	23.8	55.9	178549
373.2	259.7	50.2	OFF	23.8	55.9	179669
373.2	259.7	49.7	OFF	23.8	55.9	180810
373.2	255.8	49.3	OFF	23.8	55.9	181930
373.2	255.8	49.3	OFF	23.8	55.9	183075
373.2	252	48.8	OFF	23.8	55.9	184199
373.2	252	48.8	OFF	23.8	55.9	185338
373.2	252	48.3	OFF	23.8	55.9	186458
373.2	248.2	48.3	OFF	23.9	55.9	187597
373.2	248.2	48.3	OFF	23.9	55.9	188716
373.2	248.2	47.9	OFF	23.9	55.9	189856
373.2	248.2	47.4	OFF	23.9	55.9	190975
373.2	244.5	47.4	OFF	23.8	55.9	192115
373.2	244.5	47.4	OFF	23.8	55.9	193235
373.2	244.5	47.4	OFF	23.8	55.9	194374
373.2	244.5	47	OFF	23.8	55.9	195494
373.2	240.8	47	OFF	23.9	55.9	196633
373.2	240.8	46.5	OFF	23.9	55.9	197752
373.2	240.8	46.5	OFF	23.9	55.9	198892
Test run 11						
373.2	182.4	36.3	OFF	23.7	53.2	16445
373.2	179.6	35.9	OFF	23.7	53.2	17563
373.2	179.6	36.3	OFF	23.8	55.7	18702
373.2	179.6	36.3	OFF	23.8	55.7	19821
373.2	182.4	36.3	OFF	23.8	55.7	20961
373.2	182.4	35.9	OFF	23.8	55.7	22079
373.2	179.6	36.3	OFF	23.8	55.7	23218
373.2	182.4	36.3	OFF	23.8	55.7	24336
373.2	179.6	35.9	OFF	23.8	55.7	25474
373.2	179.6	35.9	OFF	23.8	55.7	26592
373.2	179.6	35.9	OFF	23.8	55.7	27730
367.8	179.6	35.9	OFF	23.8	55.7	28848
367.8	179.6	35.9	OFF	23.8	55.7	29986
373.2	179.6	35.9	OFF	23.8	55.7	31109
367.8	179.6	35.6	OFF	23.8	55.7	32254
373.2	176.8	35.9	OFF	23.8	55.7	33372
373.2	176.8	35.9	OFF	23.8	55.8	34511
367.8	176.8	35.6	OFF	23.8	55.8	35628
367.8	176.8	35.6	OFF	23.8	55.8	36767
373.2	179.6	35.9	OFF	23.8	55.8	37884
373.2	179.6	35.6	OFF	23.9	55.8	39021
373.2	176.8	35.6	OFF	23.9	55.8	40138
373.2	176.8	35.6	OFF	23.8	55.8	41277
373.2	176.8	35.6	OFF	23.8	55.8	42395
373.2	176.8	35.6	OFF	23.9	55.8	43533
373.2	176.8	35.6	OFF	23.9	55.8	44650
373.2	174	35.3	OFF	23.8	55.7	45787
373.2	174	35.3	OFF	23.8	55.7	46904
378.6	174	35.3	OFF	23.8	55.8	48044
373.2	176.8	35.3	OFF	23.8	55.8	49162
373.2	174	35.3	OFF	23.8	55.8	50300
373.2	174	35.3	OFF	23.8	55.8	51418
373.2	171.2	34.9	OFF	23.8	55.8	52556
373.2	174	34.9	OFF	23.8	55.8	53673
373.2	174	34.9	OFF	23.8	55.8	54810
373.2	174	35.3	OFF	23.8	55.8	55928
373.2	174	34.9	OFF	23.8	55.8	57066
373.2	174	35.3	OFF	23.8	55.8	58183
373.2	174	35.3	OFF	23.8	55.8	59323
378.6	174	35.3	OFF	23.8	55.8	60441
373.2	174	35.3	OFF	23.8	55.8	61585
373.2	176.8	35.3	OFF	23.8	55.8	62708
373.2	176.8	35.3	OFF	23.8	55.8	63846
373.2	174	35.3	OFF	23.8	55.8	64964
373.2	176.8	35.3	OFF	23.8	55.8	66102
373.2	176.8	35.3	OFF	23.8	55.8	67221
373.2	174	35.3	OFF	23.8	55.8	68360
373.2	174	35.3	OFF	23.8	55.8	69478
378.6	174	35.3	OFF	23.8	55.8	70617
373.2	174	35.3	OFF	23.8	55.8	71735
373.2	174	35.3	OFF	23.8	55.8	72873
373.2	174	35.3	OFF	23.8	55.8	73991
373.2	174	35.3	OFF	23.8	55.8	75129
373.2	174	35.3	OFF	23.8	55.8	76247
373.2	174	35.3	OFF	23.8	55.8	77385
373.2	176.8	35.6	OFF	23.8	55.8	78502
373.2	174	35.3	OFF	23.9	55.8	79642
373.2	174	35.3	OFF	23.9	55.8	80761
373.2	174	35.3	OFF	23.8	55.8	81901
373.2	174	35.3	OFF	23.8	55.8	83019
373.2	174	35.3	OFF	23.8	55.8	84158
373.2	174	35.3	OFF	23.8	55.8	85276
367.8	174	35.3	OFF	23.8	55.8	86414
367.8	174	35.3	OFF	23.8	55.8	87532
367.8	174	35.3	OFF	23.8	55.8	88672
367.8	174	35.3	OFF	23.8	55.8	89790
400.9	176.8	35.6	OFF	23.9	55.8	90928
412.4	219.8	64.3	OFF	23.9	55.8	92052
406.7	1271.7	228.6	ON	23.8	55.8	93197
454.5	4586.3	453.4	ON	23.8	55.8	94316
448.3	6635.9	513.8	ON	23.9	55.7	95456
448.3	7890.7	544.4	ON	23.9	55.7	96576
448.3	8189.9	557.2	ON	23.8	55.7	97716
448.3	8500.7	550.7	ON	23.8	55.7	98836
442.2	7509.1	513.8	ON	23.8	55.8	99975
442.2	6886.4	490.8	ON	23.8	55.8	101094
442.2	6238.9	463.8	ON	23.8	55.7	102233
442.2	5938.7	448.4	ON	23.8	55.7	103354
436.1	5653.2	428.7	ON	23.8	55.7	104495
436.1	5448.2	419.3	ON	23.8	55.7	105615
436.1	5060.5	396.7	ON	23.8	55.7	106757
454.5	4586.3	379.7	ON	23.8	55.7	107878
486.3	4420.2	371.5	ON	23.8	55.8	109018
479.8	4758.7	379.7	ON	23.8	55.8	110138
473.4	6718.3	474.4	ON	23.8	55.6	111278
473.4	9749.1	583.9	ON	23.8	55.6	112397
473.4	9274.5	544.4	ON	23.8	55.6	113537
479.8	8088.8	531.9	ON	23.8	55.6	114656
479.8	7890.7	538.1	ON	23.8	55.7	115798
473.4	7325.5	496.5	ON	23.8	55.7	116919
473.4	6718.3	474.4	ON	23.8	55.7	118060
473.4	6012.4	433.6	ON	23.8	55.7	119182
473.4	5315.8	405.6	ON	23.8	55.7	120324
473.4	5250.8	401.1	ON	23.8	55.7	121445
473.4	5123.1	392.4	ON	23.8	55.8	122592
467	4937.5	375.6	ON	23.8	55.8	123716
467	4586.3	355.7	ON	23.7	55.9	124858
467	4474.9	348.1	ON	23.7	55.9	125979
460.7	4312.9	329.8	ON	23.7	55.9	127121
460.7	4260.2	329.8	ON	23.7	55.9	128242
460.7	3861	302.9	ON	23.7	55.9	129382
460.7	3413.8	290.3	ON	23.7	55.9	130503
460.7	3170.5	275.5	ON	23.8	55.9	131643
460.7	2944.2	267	ON	23.8	55.9	132764
460.7	2944.2	272.6	ON	23.7	56	133906
460.7	2980.8	284.3	ON	23.7	56	135025
460.7	3131.6	293.4	ON	23.7	56	136167
454.5	3093.2	293.4	ON	23.7	56	137287
460.7	3017.8	290.3	ON	23.8	55.9	138429
454.5	2908.1	284.3	ON	23.8	55.9	139549
454.5	2872.4	281.3	ON	23.8	55.9	140689
460.7	2767.8	272.6	ON	23.8	55.9	141810
454.5	2569.7	256.1	ON	23.7	56	142950
454.5	2415.2	245.7	ON	23.7	56	144070
454.5	2269.7	240.7	ON	23.7	56	145212
454.5	2241.7	238.2	ON	23.7	56	146332
454.5	2106.3	228.6	ON	23.8	55.9	147472
448.3	2028.9	221.7	ON	23.8	55.9	148593
448.3	1882.2	208.6	ON	23.7	56	149733
448.3	1745.7	196.3	ON	23.7	56	150853
448.3	1639.2	190.5	ON	23.8	55.9	151994
448.3	1578.3	186.7	ON	23.8	55.9	153120
448.3	1538.9	181.1	ON	23.8	55.9	154267
448.3	1462.9	175.8	ON	23.8	55.9	155386
442.2	1408.2	170.6	ON	23.8	55.9	156526
442.2	1355.5	167.3	ON	23.8	55.9	157646
448.3	1239.5	157.6	ON	23.8	55.9	158788
442.2	1147.6	148.5	ON	23.8	55.9	159909
442.2	1048.4	141.4	ON	23.8	56	161051
442.2	969.8	133.4	OFF	23.8	56	162170
395.3	785.9	113.1	OFF	23.8	55.9	163311
395.3	735.4	109.8	OFF	23.8	55.9	164431
395.3	725.6	108.8	OFF	23.8	55.9	165570
400.9	697.1	104.7	OFF	23.8	55.9	166692
395.3	669.6	100.7	OFF	23.8	56	167834
395.3	643.1	97.8	OFF	23.8	56	168953
395.3	609.2	94.2	OFF	23.8	55.9	170091
395.3	584.9	91.5	OFF	23.8	55.9	171210
389.7	553.8	88.9	OFF	23.8	55.9	172349
395.3	538.8	86.4	OFF	23.8	55.9	173469
395.3	517	84	OFF	23.8	55.9	174609
395.3	502.9	82.4	OFF	23.8	55.9	175728
395.3	482.4	80.8	OFF	23.8	55.9	176867
395.3	469.2	79.3	OFF	23.8	55.9	177987
389.7	469.2	78.5	OFF	23.8	55.9	179127
395.3	469.2	78.5	OFF	23.8	55.9	180247
389.7	456.3	77	OFF	23.8	55.8	181387
389.7	443.7	74.9	OFF	23.8	55.8	182505
400.9	443.7	76.3	OFF	23.8	55.9	183650
400.9	437.5	76.3	OFF	23.8	55.9	184775
395.3	431.4	74.9	OFF	23.8	55.9	185914
395.3	419.4	73.5	OFF	23.8	55.9	187033
400.9	402	72.1	OFF	23.8	56	188173
395.3	396.3	70.7	OFF	23.8	56	189290
395.3	385.2	68.7	OFF	23.8	56	190431
395.3	374.3	68.1	OFF	23.8	56	191550
395.3	369	66.8	OFF	23.8	56	192692
395.3	358.6	66.8	OFF	23.8	56	193811
395.3	348.4	64.9	OFF	23.8	56	194952
395.3	343.4	63.7	OFF	23.8	56	196070
395.3	338.5	63.1	OFF	23.8	56	197210
395.3	333.6	61.9	OFF	23.8	56	198329
395.3	324	60.7	OFF	23.8	56	199469
395.3	319.4	60.2	OFF	23.8	56	200588
395.3	314.7	59.6	OFF	23.8	56	201728
395.3	310.2	58.5	OFF	23.8	56	202846
395.3	305.7	57.9	OFF	23.8	56	203986
395.3	301.2	57.4	OFF	23.8	56	205105
395.3	296.8	56.8	OFF	23.8	56	206247
395.3	292.5	56.8	OFF	23.8	56	207365
395.3	288.2	55.8	OFF	23.8	56	208507
395.3	288.2	54.7	OFF	23.8	56	209626
395.3	283.9	54.2	OFF	23.8	56	210767
395.3	283.9	53.7	OFF	23.8	56	211886
395.3	279.8	53.7	OFF	23.8	56	213026
389.7	275.6	53.2	OFF	23.8	56	214149
395.3	275.6	52.7	OFF	23.8	56	215295
389.7	271.6	52.2	OFF	23.8	56	216414
389.7	267.6	51.7	OFF	23.8	56	217554
389.7	267.6	51.7	OFF	23.8	56	218672
389.7	271.6	52.2	OFF	23.8	56	219813
395.3	267.6	51.7	OFF	23.8	56	220933
389.7	267.6	51.2	OFF	23.8	56	222074
395.3	263.6	50.7	OFF	23.8	56	223193
395.3	263.6	50.7	OFF	23.8	56	224336
389.7	255.8	49.7	OFF	23.8	56	225455
389.7	259.7	49.3	OFF	23.8	56	226596
389.7	263.6	49.3	OFF	23.8	56	227715
